# Validation of the Sysmex XN‐V hematology analyzer for canine specimens

**DOI:** 10.1111/vcp.12936

**Published:** 2021-06-21

**Authors:** Margot Grebert, Fanny Granat, Jean‐Pierre Braun, Quentin Leroy, Nathalie Bourgès‐Abella, Catherine Trumel

**Affiliations:** ^1^ Département des Sciences Cliniques des animaux de compagnie et de sport Université de Toulouse ENVT Toulouse France; ^2^ CREFRE Université de Toulouse INSERM ENVT UPS Toulouse France; ^3^ CRCT Université de Toulouse INSERM UMR 1037 Toulouse France; ^4^ Animal and Comparative Clinical Pathology Toulouse France

**Keywords:** blood, comparison study, dog, method validation

## Abstract

**Background:**

The Sysmex XN‐V is derived from the new Sysmex XN series of human hematology analyzers. The main changes from the previously validated XT‐2000iV analyzer include an optic‐fluorescent analysis for platelets and nucleated RBC count.

**Objective:**

We aimed to validate the Sysmex XN‐V for canine blood according to American College for Veterinary Clinical Pathology and International Council for Standardization in Hematology recommendations.

**Materials and Methods:**

Canine EDTA blood specimens and quality control material were analyzed on the Sysmex XN‐V to evaluate imprecision, bias, linearity, a comparison with the XT‐2000iV analyzer, interference effects, carry‐over, and stability. We also verified previously established Sysmex XT‐2000iV reference intervals (RIs).

**Results:**

Imprecision and bias were low (<5%) for most variables. Observed total error was lower than allowable total error for most measured variables except lymphocytes and monocytes. Visually determined linearity was excellent for all variables, except for lymphocytes. The correlation between the XN‐V and XT‐2000iV analyzers was high (>0.93) for all variables except MCHC and reticulocyte indices. Correlations between the Sysmex XN‐V and manual differential counts were good for neutrophils and eosinophils, acceptable for lymphocytes, and fair for monocytes. Hemolysis, lipemia, and to a lesser extent icterus, had significant effects on measured hemoglobin concentration and associated variables. Carry‐over was not visually observed for any variable. Changes in the Sysmex XN‐V measurements after storage at 4℃ and 24℃ were similar to those described for the Sysmex XT‐2000iV analyzer. The previously established Sysmex XT‐2000iV RIs can be used to interpret results from the Sysmex XN‐V analyzer for most variables except red blood cell distribution width and mean platelet volume.

**Conclusions:**

The performance of the Sysmex XN‐V analyzer was excellent and compared favorably with the Sysmex XT‐2000iV analyzer.

## 1 INTRODUCTION

Large, automated hematology analyzers with multispecies software are of great importance in veterinary laboratories and industry. The Sysmex XN‐V is a new analyzer, derived from the Sysmex XN series for analyzing human blood. The main changes from the XT‐2000iV^1^, ^2^ include an optic‐fluorescent analysis for platelets (PLT‐F channel using an oxazine‐based fluorescent dye), a nucleated RBC (NRBC) count, and new software for the data analysis of 11 animal species.

Validation of a new instrument is recommended before its implementation. According to the American Society of Veterinary Clinical Pathology (ASVCP), validation includes an evaluation of linearity, repeatability, reproducibility, method comparison, hemolysis, lipemia, and bilirubin interference studies, a recovery study, reference interval (RI) determinations, and a detection limit study.^3^, ^4^, ^5^ According to the International Council for Standardization in Hematology (ICSH), evaluation should also include carry‐over and specimen stability, but not recovery and detection limit studies.^6^

The purpose of this study was to validate the Sysmex XN‐V hematology analyzer for canine blood analyses according to ASVCP and ICSH recommendations. We also compared the Sysmex XN‐V RIs with those of the XT‐2000iV hematology analyzer, which had been previously validated in canine blood samples.^1^, ^2^, ^7^, ^8^

## 2 MATERIALS AND METHODS

This study was approved by the “Science et Santé animale” Ethics Committee (N° SSA_2019_002).

### 2.1 Specimens

Experiments were performed on fresh canine K3‐EDTA blood samples (Venosafe, Terumo) and two levels of manufacturer's quality control material (XN‐CHECK QC1 & QC2; Sysmex Europe GmbH). Fresh blood specimens were received for the hematologic evaluation at the Laboratoire Central de Biologie Médicale (Central Laboratory of Clinical Pathology) to perform patient diagnostic workups or health checkups at the National Veterinary School of Toulouse. As the patients were presented to various disciplines (internal medicine, surgery, emergency, intensive care, oncology department, and routine ambulatory), specimens were obtained from dogs with different diseases or conditions, thus covering a wide range of results and a wide variety of blood disorders. One labeled air‐dried blood smear was prepared within 1 hour of blood sampling and stained with a May‐Grünwald/Giemsa automatic stainer (Aerospray Hematology Slide Stainer Cytocentrifuge 7150, Wescor), and was then stored before microscopic evaluation. Prior to hematologic analysis, blood specimens were kept at room temperature (24℃), placed on an agitator (Specie mix, Drew Scientific Inc) for 20 minutes, and then gently inverted to ensure homogenization. Specimens with visible or microscopic clots were excluded. Measurements were performed within 2 hours after sampling with the Sysmex XN‐V and Sysmex XT‐2000iV (Sysmex Corporation), according to the manufacturer's instructions, using settings for “quality control material” or “dog” (Sysmex XN‐V software v.00‐05; Sysmex XT‐2000iV software v.00‐13).

### 2.2 Variables analyzed

The following variables were analyzed with both analyzers: impedance and optical RBC counts (RBC‐I, RBC‐O), hematocrit (HCT), hemoglobin concentration (HGB), mean corpuscular volume (MCV), mean corpuscular hemoglobin (MCH), mean corpuscular hemoglobin concentration (MCHC), red cell distribution width standard deviation and coefficient of variation (RDW‐SD and RDW‐CV, respectively), impedance, optical and fluorescence platelet counts (PLT‐I, PLT‐O, PLT‐F, respectively), mean platelet volume (MPV), plateletcrit (PCT), platelet distribution width (PDW), platelet large cell ratio (P‐LCR), immature platelet fraction (IPF), reticulocyte count and percentage (RET), and low‐, medium‐, and high‐reticulocyte fluorescence ratio (LFR, MFR, HFR), immature reticulocyte fraction (IRF), reticulocyte hemoglobin equivalent (RET‐He), nucleated red blood cell count (NRBC), mature RBC hemoglobin equivalent (RBC‐He), WBC, counted on the white cell nucleated (WNR) or WBC differential (WDF) channel, and WBC differential count (DIFF). Manual 200‐cell WBC DIFF and NRBC counts (expressed as the number of NRBCs per 100 leukocytes) were performed by the first author (E200 microscope, Nikon, Kobe, Japan) and packed cell volumes (PCVs) were measured by the centrifugation of blood in microhematocrit tubes (Haematokrit 210, Hettich).

### 2.3 Method validation and comparison study

Repeatability was evaluated from 20 consecutive repeats of a randomly selected canine specimen analyzed within 2 hours of sampling. Reproducibility was evaluated by duplicate morning and afternoon measurements of the manufacturer's quality control material (QC1 & QC2) for five consecutive days.

Linearity was only tested on analytes expressed as concentrations or counts and not for those expressed as indices or percentages. Linearity was evaluated by analyzing four repeats of a 5‐point (0%, 25%, 50%, 75%, 100%) serial dilution of a fresh canine EDTA blood specimen with the Sysmex diluent solution, DCL (Cellpack DCL, Sysmex Europe GmbH). Results of diluted specimens below the lower limit of measurement were considered as 0.

The measurements by the two analyzers were compared by paired analyses of fresh EDTA blood specimens less than 2 hours after blood collection, with a delay of less than 30 minutes between measurements. Blood specimens with platelet clumps visualized on the blood smear or flagged by the analyzer were excluded. Basophils were not included in the comparison because the basophil count has been reported to be unreliable.^9^

Each result was classified according to canine RIs previously established for the Sysmex XT‐2000iV,^7^ and the number of results within, above, or under the reference limits by the two analyzers was counted.

**FIGURE 1:**
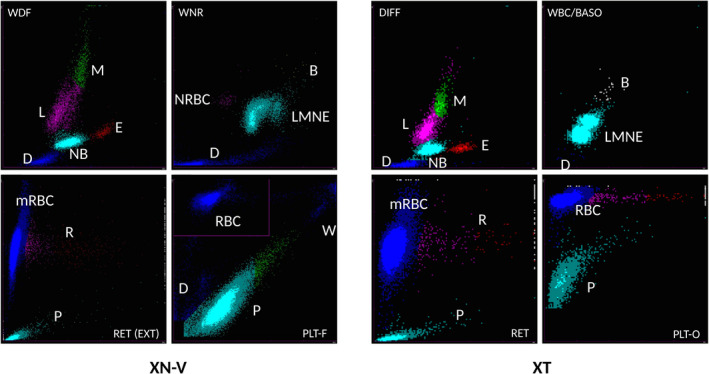
Cell identification comparisons on Sysmex XN‐V and the Sysmex XT‐2000iV scattergrams according to the manufacturer. WBC differential scattergrams (WDF and DIFF); WBC count scattergrams (WNR and WBC/BASO); reticulocyte scattergrams (RET(EXT) and RET); platelet scattergrams (PLT‐F and PLT‐O). B, basophils; D, debris; DIFF, WBC differential scattergram; L, lymphocytes; M, monocytes; mRBC, mature RBC; N, neutrophils; NRBC, nucleated red blood cells; P, platelets; PLT‐F, platelet scattergram with optic‐fluorescent analysis; PLT‐O, platelet scattergram with optic analysis; R, reticulocytes; RBC, red blood cells; RET, reticulocyte scattergram; RET(EXT), reticulocyte extended scattergram; W, WBC; WBC, white blood cells; WBC/BASO, WBC scattergram with the basophil channel; WDF, WBC differential; WNR, white cell nucleated

Differences between the XN‐V and XT‐2000iV scattergrams are presented in Figure 1. The flags and scattergrams were checked and investigated when a clinically relevant discrepancy between the manual and XN‐V leukocyte differential count was observed.

The effects of hemolysis, hyperbilirubinemia, and hyperlipemia were assessed using previously published protocols.^10^ EDTA blood specimens from three control dogs with no visible plasma color abnormality were aliquoted. Increasing concentrations of commercial hemoglobin (Bovine Hemoglobin, Sigma‐Aldrich), bilirubin (Bilirubin purum >95%, Sigma‐Aldrich), or lipids (Intralipid 20%, Sigma‐Aldrich) solutions were mixed into the aliquots from each dog, and triplicate analyses were then performed with the Sysmex XN‐V. The final concentrations of each interfering substance were chosen to simulate slight, moderate, severe, and extreme hemoglobinemia (hemoglobin concentrations of 0.62, 1.25, 2.50, and 5.00 g/L), icterus (total bilirubin concentrations, 0.02, 0.04, 0.08, and 0.15 g/L), and lipemia (triglycerides concentrations, 1.25, 2.50, 5.00, and 10.00 g/L).

Carry‐over was evaluated using a series of five triplicate analyses with high‐level and then low‐level quality control materials.^6^ Stability was determined using ten randomly selected fresh EDTA specimens stored at room temperature and 4℃. Duplicate analyses were performed immediately and 2, 4, 8, 24, 48, and 72 hours later. The specimens stored at 4℃ were analyzed after rewarming at room temperature for 20 minutes and were thus analyzed after the room temperature specimens.

The canine hematologic RIs previously established for the Sysmex^7^ were verified according to ASVCP and IFCC recommandations^4^, ^11^, ^12^, ^13^ in 20 EDTA blood specimens from healthy dogs selected on the basis of history and physical examination.

### 2.4 Statistical analysis

Repeatability and reproducibility were evaluated as the coefficient of variation, CV = (SD/Mean)∗100. Bias was calculated from manufacturer's data as Bias = ([expected − obtained] values/expected values)∗100. Observed total error was then calculated as TE_obs_ = Bias + 2∗CV. When TE_obs_ was higher than the allowable total error (TE_a_), the quality goal index (QGI) was calculated as recommended by the ASVCP; QGI = Bias/(1.5∗CV). Linearity was tested visually and with linear regression analysis. A comparison of results was based on Spearman's correlation, Passing‐Bablok agreement analysis, and difference diagrams of the results obtained by the XN‐V and the XT‐2000iV, or by the XN‐V and the manual methods for WBC differential count, HCT, and NRBC count.^5^ Effects of interferences were evaluated by visual inspection of results and repeated measures ANOVA, and when significant, followed by Dunnett's test. Effects of carry‐over were examined visually and tested by within‐ and between‐series ANOVA. The effects of storage time and temperature were assessed by comparing results at all durations of storage to initial measurements by ANOVA, and when results were significant, followed by Tukey's honestly significant difference test. Sysmex XT‐2000iV RIs were verified according to the Clinical and Laboratory Standards Institute (CLSI) and ASVCP guidelines.^11^, ^12^, ^13^ Statistical analyses were performed with Excel (Microsoft), Analyse‐It (Analyse‐It), and Systat (Systat Software). The level of significance was set at *P* < 0.05.

## 3 RESULTS

Given the numerous variables, we have chosen to report the full results for RBC, WBC, PLT, and RET in tables and figures within the manuscript, while detailed results for the other variables are given in the supplementary material.

### 3.1 Imprecision ‐Bias ‐ TE_obs_

Results of repeatability with canine EDTA blood and within‐ and between‐run of quality control material with the Sysmex XN‐V analyzer are shown in Tables 1 and S1. Imprecision and bias were <5% for most variables. Imprecision was notable for lymphocyte, monocyte, and eosinophil counts, reticulocyte and PLT indices, and PLT‐I and PLT‐O counts, and bias was >5% for monocyte counts, PLT counts, and reticulocyte indices. TE_obs_ was <20% for most measured variables, excluding lymphocyte, monocyte, and eosinophil counts, and higher for the PLT and RET indices. TE_obs_ was lower than TE_a_ for all variables except lymphocyte and monocyte counts. The latter QGI were <0.8 and >1.2, respectively (Table S1).

**TABLE 1:** Imprecision, bias, and total error of RBC‐I, WBC, PLT‐F, and RET measurements with the Sysmex XN‐V hematology analyzer using quality control solutions (QC1 and QC2) and a randomly selected canine specimen

Variable	Unit	QC1 solution					QC2 solution					Canine blood		
		Target	Repeat	Reprod	Bias %	TE_obs_ ^a^ %	Target	Repeat	Reprod	Bias %	TE_obs_ ^a^ %	Mean value	Repeat CV %	TE_a_ ^a^ %
			CV %					CV %						
RBC‐I	10^12^/L	2.32	0.55	1.07	1.88	4.02	4.36	0.55	0.83	1.23	2.89	5.59	1.08	10
WBC	10^9^/L	3.06	1.54	2.00	−0.21	4.21	6.86	0.72	1.05	−1.46	3.56	7.59	1.36	15
PLT‐F	10^9^/L	85	3.05	3.45	1.18	8.08	255	0.96	2.11	10.45	14.67	203	1.74	20
RET	10^9^/L	134	2.13	2.55	−4.27	9.37	112	2.78	2.85	−2.97	8.67	11.8	5.03	20

Abbreviations: CV% = SD/Mean*100; PLT‐F, fluorescence platelet count; QC1, low‐level quality control material; QC2, within reference interval quality control material; RBC‐I, impedance RBC count; Repeat, repeatability; Reprod, reproducibility; RET, reticulocyte count; TE_a_, allowable total error; TE_obs_, observed total error.

^a^ TE_a_ and TE_obs_ were calculated and reported according to Nabity et al.^16^

### 3.2 Linearity

**FIGURE 2:**
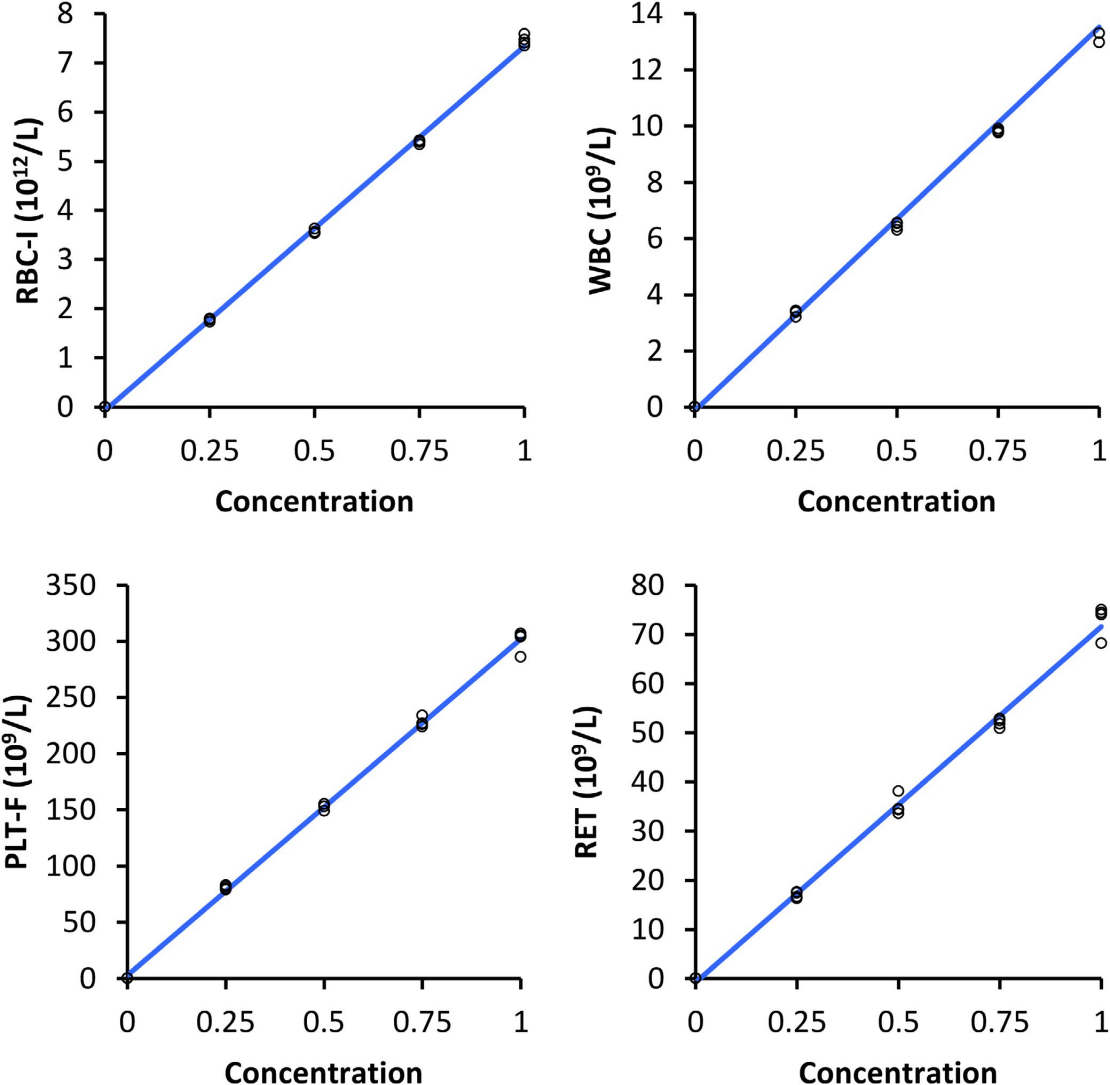
Measurements of canine RBC‐I, WBC, PLT‐F, and RET with the Sysmex XN‐V analyzer after dilution (concentration of undiluted specimen normalized to 1). PLT‐I, impedance platelet count; RBC‐I, impedance RBC count; RET, reticulocyte count

Linearity could not be tested for eosinophils, as the counts were very low. Visually determined linearity was excellent for all variables, except lymphocyte counts (Figure 2; Figure S1), and even when linearity held, a polynomial fit was better. However, maximum differences from linearity were <5.5%, excluding the monocyte count (16.1%).

### 3.3 Comparison of XN‐V results with XT and manual results

**FIGURE 3:**
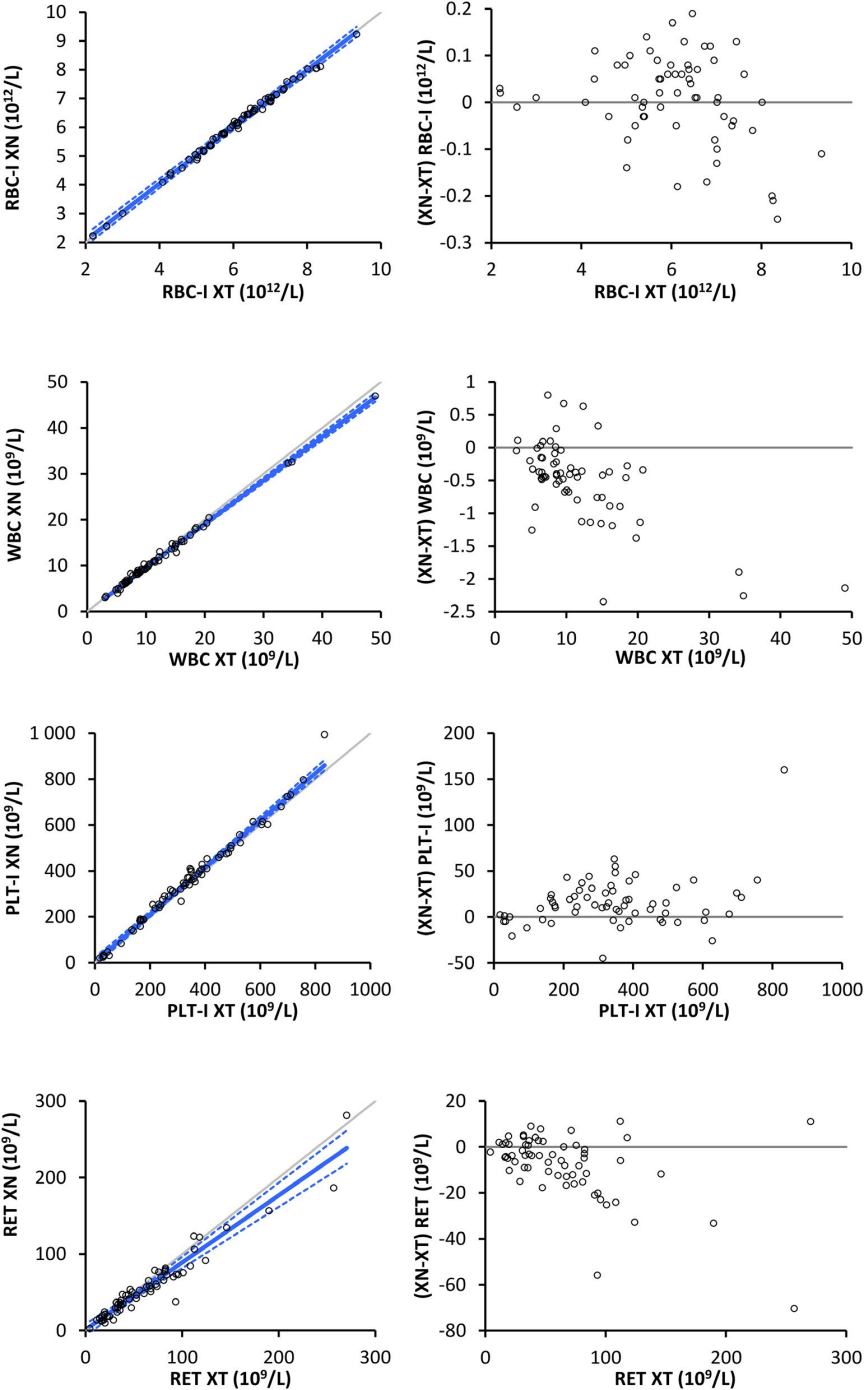
Passing‐Bablok plots (left) and difference diagrams (right) for RBC‐I, RET, WBC, and PLT‐I between the Sysmex XN‐V and Sysmex XT‐2000iV hematology analyzers. In the Passing‐Bablok plots, the thin gray line is identity (*y* = *x*); and the blue line is the regression curve with 95% confidence intervals. RBC‐I, impedance RBC count; PLT‐I, impedance platelet count; RET, reticulocyte count

This part of the study included 20 healthy dogs and 44 ill dogs. Hematologic disorders included anemia (15 dogs), polycythemia (3), leukocytosis (3), leukopenia (3), thrombocytopenia (7), thrombocytosis (7), leukemia (2), macrocytosis (6), or microcytosis (4), reticulocytosis (7), the presence of NRBC (11), suspected immune‐mediated hemolytic anemia based on regenerative anemia and spherocytosis (1 dog), hemoparasites (ie, piroplasmosis [1 dog] and microfilariosis [1]), and leukocyte abnormalities, such as a left shift (9), toxic change (2), reactive lymphocytes (14), activated monocytes (9), basophilia (6), or mastocytemia (2). To compare the HCT results between analyzers, one specimen was excluded because the manual PCV was unavailable. One specimen was excluded from the comparison between the Sysmex XN‐V and the manual differential leukocyte count because the slide was damaged. A comparison between the two analyzers is reported in Table 2, Table S2, Figure 3 and Figure S2. The correlation between the XN‐V and the XT‐2000iV analyzers was high (>0.93) for all variables except for MCHC (*r* = 0.78) and RET indices (LFR, *r* = 0.69; MFR, *r* = 0.43; HFR, *r* = 0.34; IRF, *r* = 0.69; RET‐He, *r* = 0.80). Passing‐Bablok regression equation slopes and intercepts were close to 1 and 0, respectively, for most variables. Results for the comparison between the Sysmex XN‐V analyzer and manual 200‐cell differential leukocyte counts, NRBC counts, and PCVs measured by centrifugation are shown in Table 3. The correlation between the HCT value determined with the Sysmex XN‐V and the manual PCV measurement was excellent (*r* = 0.96). The correlation between the Sysmex XN‐V and manual differential counts was good for neutrophils and eosinophils (*r* = 0.84 and 0.86, respectively), acceptable for lymphocytes (*r* = 0.76), and fair for monocytes and NRBC (*r* = 0.45 and 0.57, respectively) (Table 3).

**TABLE 2:** Comparison of canine blood RBC‐I, WBC, PLT‐I, and RET measurements (n = 64) using the Sysmex XT‐2000iV and Sysmex XN‐V hematology analyzers (95% confidence intervals of Spearman's correlation coefficient *r* and Passing‐Bablok equation coefficients between brackets)

	Median (min to max)			Spearman's *r*	Passing‐Bablok equation XN = *a*·XT + *b*	
	XT	XN	XN‐XT		*a*	*b*
RBC‐I, 10^12^/L	6.13 (2.00‐9.00)	6.16 (2.2‐9.2)	0.01 (−0.25 to 0.19)	1.00 (0.99‐1.00)	0.99 (0.97‐1.00)	0.10 (−0.01 to 0.23)
WBC^a^, 10^9^/L	9.55 (3.03‐49.05)	9.13 (2.98‐46.91)	−0.42 (−2.35 to 0.80)	0.99 (0.99‐1.00)	0.95 (0.93‐0.97)	0.03 (−0.17 to 0.24)
PLT‐I, 10^9^/L	340 (38‐834)	350 (20‐994)	19 (−36 to 116)	0.99 (0.98‐0.99)	1.03 (1.00‐1.06)	3.32 (−3.25 to 12.8)
RET, 10^9^/L	52.6 (4.4‐270.4)	47.7 (2.2‐281.5)	−4.4 (−70.4 to 11.2)	0.95 (0.92‐0.97)	0.88 (0.80‐0.97)	1.16 (−2.76 to 4.43)

Abbreviations: max, maximum; min, minimum; PLT‐I, impedance platelet count; RBC‐I, impedance RBC count; RET, reticulocyte count, XN, Sysmex XN‐V; XT, Sysmex XT‐2000iV.

^a^ One pair of outliers deleted (WBC_XT_: 209·10^9^/L; WBC_XN_: 192·10^9^/L).

**TABLE 3:** Passing‐Bablok agreement between the Sysmex XN‐V results and the manual measurements in canine blood specimens (n = 63) (95% confidence intervals of Spearman's coefficient *r* and Passing‐Bablok equation coefficients between brackets)

	Median (min to max)			Spearman's *r*	Passing‐Bablok equation XN = *a*·Man + *b*	
	Manual	XN	Manual‐XN		*a*	*b*
Neutrophils, %	71.8 (4.5‐96.0)	69.4 (0.0‐90.4)	−2.8 (−77.1 to 9.5)	0.84 (0.75‐0.90)	1.00 (0.92‐1.12)	−2.85 (−11.37 to 3.90)
Lymphocytes, %	16.5 (0.0‐93.5)	16.4 (5.1‐87.0)	1.0 (−27.4 to 74.7)	0.76 (0.63‐0.85)	0.82 (0.72‐0.94)	3.64 (1.81‐4.69)
Monocytes, %	6.5 (1.5‐24.5)	7.4 (2.3‐33.1)	1.1 (−4.2 to 31.1)	0.45 (0.23‐0.63)	1.00 (0.82‐1.33)	1.10 (−1.17 to 2.23)
Eosinophils, %	2.5 (0.0‐12.5)	2.7 (0.0‐18.0)	0.2 (−1.5 to 6.0)	0.86 (0.78‐0.91)	1.10 (0.95‐1.45)	0.10 (−0.22 to 0.40)
HCT, L/L	0.400^a^ (0.147‐0.592)	0.404 (0.147‐0.593)	0.000 (−0.022 to 0.028)	0.96 (0.93‐0.98)	1.04 (1.00‐1.10)	−0.01 (−0.03 to 0.01)
NRBC, 10^9^/L	0.00 (0.00‐1.06)	0.01 (0.00‐1.12)	0.00 (−0.08 to 0.32)	0.57 (0.37‐0.71)	1.02 (0.49‐1.77)	0.00 (0.00‐0.00)

Abbreviations: HCT, hematocrit; Manual‐XN, difference between manual and XN measurements; max, maximum; min, minimum; NRBC, nucleated RBC count; XN, Sysmex XN‐V.

^a^ Packed cell volume.

For 14/64 cases, flags were observed on the result sheets as star symbols next to the numerical result and/or text messages indicating an abnormal WBC, RET, RBC, and/or PLT scattergram or distribution. Platelet, WBC, RBC, and RET flags were observed in 9, 7, 2, and 3/64 cases, respectively. In one case, several flags were observed.

**FIGURE 4:**
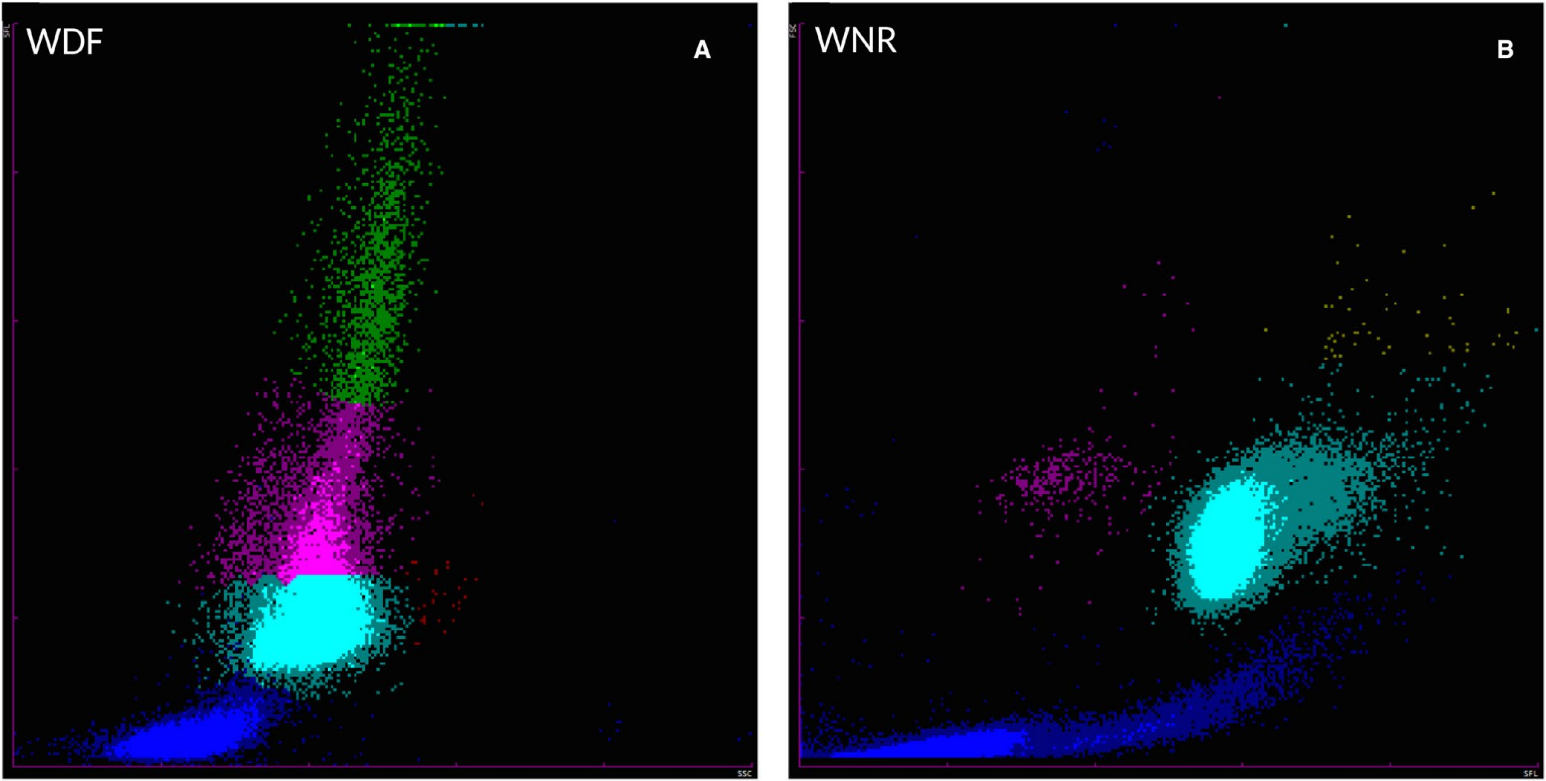
WDF (A) and WNR (B) scattergrams from the Sysmex XN‐V analyzer for a canine blood specimen with a high percentage of band cells based on the manual differential count. (A) On the WDF scattergram, a well‐defined but extended population was observed at the neutrophils position and identified as neutrophils (turquoise dots) and lymphocytes (pink dots), despite a lack of cluster at the normal lymphocytes position. The WBC count was 32.3 × 10^9^ cells/L. The neutrophil percentages were 74.9% (XN) and 90.5% (manual; with 68.5% segmented neutrophils and 22% band cells); the lymphocytes percentages were 18.8% (XN) and 4.0% (manual); the monocytes percentages were 6.0% (XN) and 5.5% (manual); and the eosinophils percentages were 0.1% (XN) and 0.0% (manual). A flag was reported for neutrophils and lymphocytes, as well as an error message “WBC Abn Scattergram.” In this case, flag and/or error messages were also observed for PLT and RET. (B) WNR scattergram of the same dog. PLT, platelet count; RET, reticulocyte count; WDF, WBC differential; WNR, white cell nucleated

**FIGURE 5:**
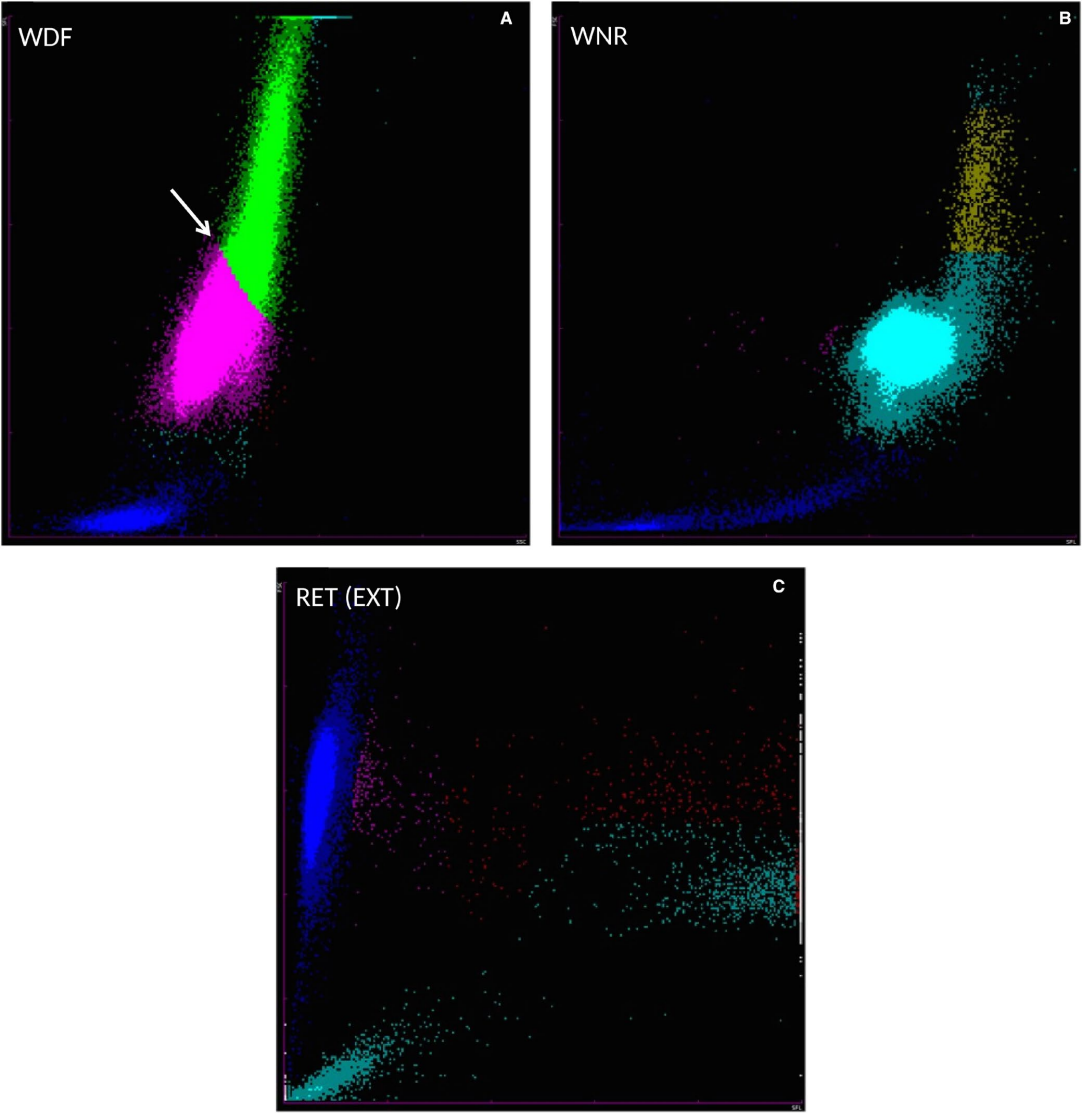
WDF (A), WNR (B), and RET‐EXT (C) scattergrams from the Sysmex XN‐V analyzer for a blood specimen from a dog with acute leukemia. (A) On the WDF scattergram, a large cloud is present at the location of the lymphocytes, neutrophils, and monocytes populations, and an arbitrary separation was performed by the analyzer (white arrow). (B) On the WNR scattergram, the cluster of leukocytes extended to the upper right part of the scattergram. (C) On the RET‐EXT scattergram, the reticulocyte clouds were characterized by a gap between mature reticulocytes (pink dots), and a population of high‐fluorescence particles (red and turquoise dots) suspected to be leukemic cells and not platelets (turquoise dots) or high‐fluorescence RET (red dots). The WBC count was 191.9 × 10^9^ cells/L. The neutrophil percentages were 0.0% (XN) and 4.5% (manual); the lymphocytes percentages were 66.1% (XN) and 0.0% (manual); the monocytes percentages were 33.1% (XN) and 2.0% (manual); and the eosinophils percentages were 0.0% (XN and manual). Blastic cells (93.5%) were only revealed by manual counting. A flag was reported for monocytes and lymphocytes results, as well as an error message “WBC Abn Scattergram”. In this case, flag and/or error messages were also observed for PLT and RET. PLT, platelet count; RET, reticulocyte count; RET(EXT), reticulocyte extended; WDF, WBC differential; WNR, white cell nucleated

Major discrepancies between the Sysmex XN‐V and manual differential counts were observed in nine cases, while flags were raised in six of these. Scattergrams showed two main errors in seven cases mainly associated with a high percentage of band cells based on the manual differential counts, and neutrophils recognized by the XN‐V as neutrophils and lymphocytes, despite a well‐defined but extended population observed in the neutrophil position with no dot clusters in the normal lymphocyte positions (Figure 4). In another case of acute leukemia, suspected to be acute lymphoid leukemia, a large cloud was observed in the neutrophil, lymphocyte, and monocyte positions, and an arbitrary separation was performed by the XN‐V analyzer (Figure 5).

### 3.4 Interferences

**FIGURE 6:**
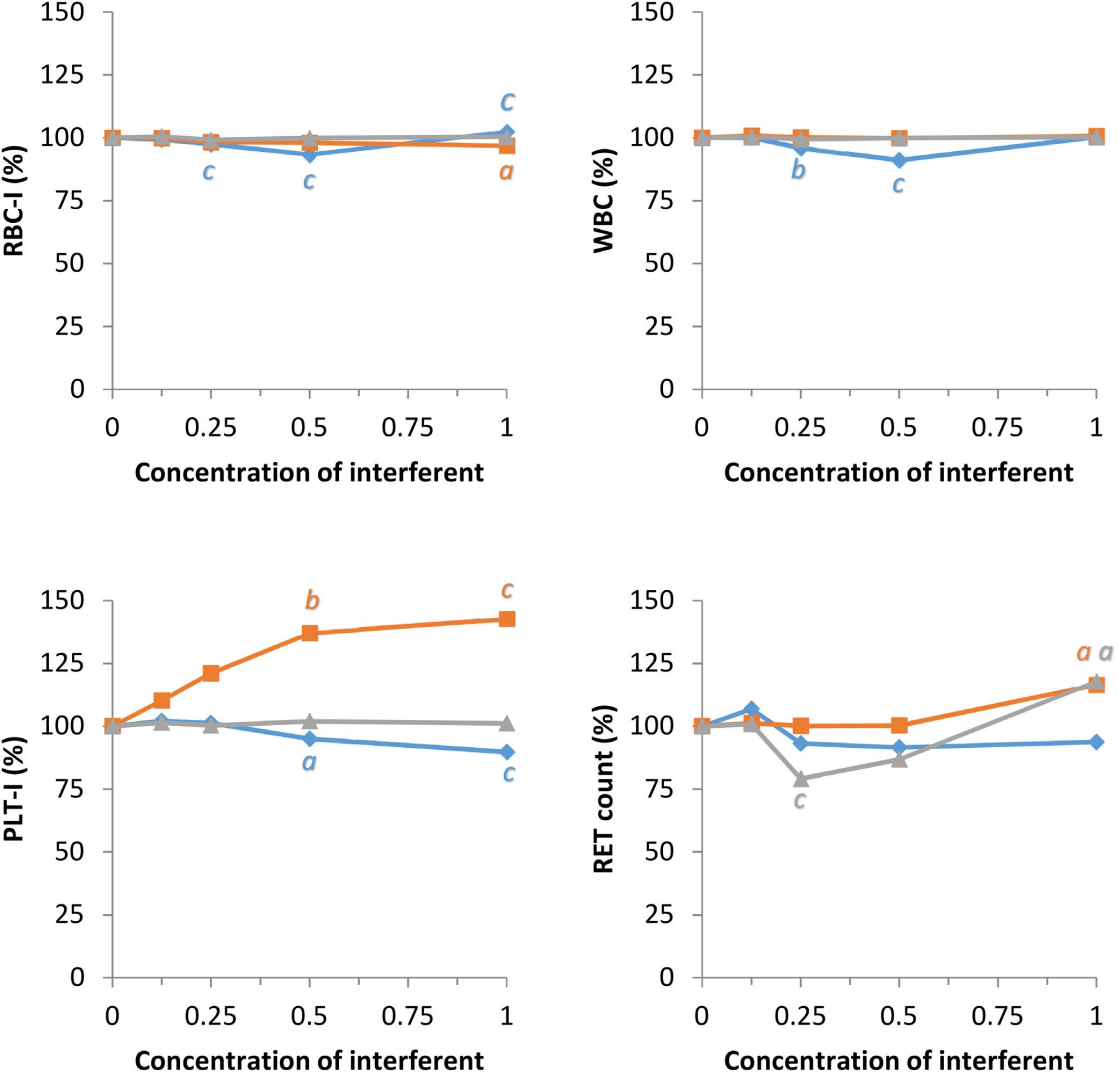
Effects of hemolysis (blue), lipemia (orange), and icterus (gray) on canine RBC‐I, WBC, and PLT‐I and RET measurements with the Sysmex XN‐V (maximum concentrations of hemoglobin: 5 g/L; triglycerides: 10 g/L; bilirubin: 0.15 g/L; changes as % difference from native specimen); a, b, c: comparison with the native specimen using Dunnett's test, *P* <.05; 0.01; 0.001. PLT‐I, impedance platelet count; RBC‐I, impedance RBC count; RET, reticulocytes

Results for the effects of hemolysis, lipemia, and icterus on hematology variables are shown in Figure 6 and Figure S3. HGB, MCH, and MCHC were significantly increased with slight (for HGB) or severe (for MCH, MCHC) hemoglobinemia. The maximum differences were <7.1%. HGB, MCH, and MCHC were significantly increased by the addition of lipids, with maximum differences of 19.7%, 23.4%, and 19.5%, respectively, observed in cases with extreme lipemia. Lipemia had a significant and proportional effect on PLT‐I and PLT‐O counts, with a maximum difference of +42.7% and +38.5%, respectively. Icterus had a significant but slight effect on HGB and a markedly intense effect on PLT‐O with maximum increases of +1.6% and +54.4%, respectively.

### 3.5 Carry‐over

**FIGURE 7:**
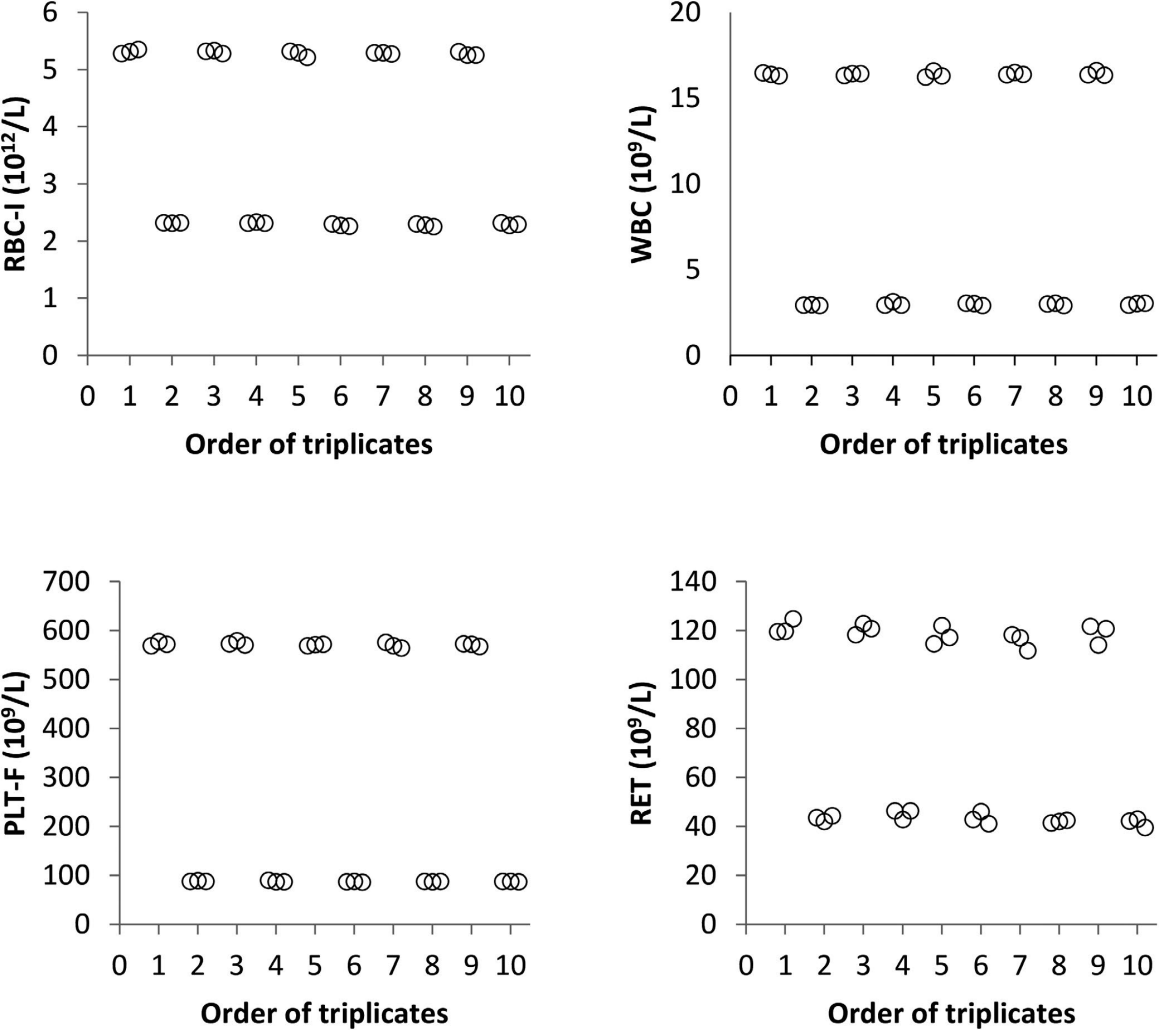
Effects of carry‐over on RBC‐I, WBC, PLT‐F, and RET measurements with the Sysmex XN‐V (Five repeats of triplicates using the manufacturer's high and low control solutions). PLT‐F, fluorescence platelet count; RBC‐I, impedance RBC count; RET, reticulocytes

Carry‐over was not visually observed for any variable (Figure 7; Figure S4). However, statistically significant differences between triplicates (*P* < 0.05) were observed. They were ≤2.1% for low HCTs, ≤0.7% for high and low MCVs, ≤3.3% for high MCHC values, ≤0.6% for high RBC‐He values, and ≤1.4% for low RET‐He values.

### 3.6 Stability

The changes in Sysmex XN‐V measurements after storage at 4℃ and 24℃ are presented in Tables 4 and S4. Mean measurements for the RBC variables after storage of up to 72 hours were significantly different from *T*_0_ except for RBC counts. The effect of storage temperature was significantly different for all variables, except RBC‐O, HGB, PLT‐O, and PLT‐F. Compared with *T*_0_, the maximum differences after 72 hours of storage were an increase in the HCT (+18%) and MCV (+25%), and a decrease in the MCHC (−15%) at 24℃. Hemoglobin remained stable for up to 72 hours under both storage conditions. A significant and time‐dependent decrease in PLT‐I, PLT‐O, and PLT‐F was observed with a maximum difference of −47% (at 4℃), −28% (at 4℃), and −41% (at 24℃), respectively, after 72 hours of storage. No significant difference between storage at 4℃ and 24℃ was observed for PLT‐O and PLT‐F.

**TABLE 4:** Percent changes in RBC‐I, WBC, PLT‐F, and RET measurements on the Sysmex XN‐V analyzer for 10 canine EDTA blood specimens according to temperature and duration of storage

Variable	*T*_0_	Temperature		Effects of storage (h): comparison to *T*_0_						
		*P*		*P*	2	4	8	24	48	72
RBC‐I	3.92‐7.55 10^12^/L	<0.001	4	0.008	100	99	100	100	99	99
					*0.994*	*0.738*	*0.874*	*1.000*	*0.087*	*0.358*
			24	0.998	100	100	99	99	97	96
					*0.997*	*0.967*	*0.873*	*0.183*	*<0.001*	*<0.001*
WBC	6.69‐29.06 10^9^/L	<0.001	4	0.143	101	101	101	101	101	102
					—	—	—	—	—	—
			24	<0.001	*99*	*103*	*99*	*99*	*97*	*95*
					*0.954*	*0.400*	*0.958*	*0.252*	*<0.001*	*<0.001*
PLT‐F	240‐882 10^9^/L	0.513	4	<0.001	94	95	90	83	72	65
					*0.881*	*0.794*	*0.178*	*<0.001*	*<0.001*	*<0.001*
			24	<0.001	97	90	91	85	68	59
					*1.000*	*0.992*	*0.911*	*0.417*	*<0.001*	*<0.001*
RET	23.0‐194.7 10^9^/L	0.004	4	<0.001	99	98	98	104	103	109
					*0.998*	*0.999*	*1.000*	*0.461*	*0.883*	*<0.001*
			24	<0.001	100	102	99	101	110	119
					*1.000*	*1.000*	*0.999*	*1.000*	*0.147*	*0.002*

Note

*T*_0_, range of measurements at *T*_0_; *P*, ANOVA of the effects of temperature and duration of storage; 2‐72, effects of duration of storage: mean of percent of *T*_0_; in italics, comparison to *T*_0_ by Tukey's HSD test.

Abbreviations: PLT‐F, fluorescence platelet count; RBC‐I, impedance RBC count; RET, reticulocyte count.

### 3.7 Reference intervals

For most variables except RDW‐SD, RDW‐CV, MPV, and HFR, the acceptance criterion of ≤2 for the 20 values fell inside the previously established RIs for the Sysmex XT‐2000iV when the confidence interval of the limits was taken into account.^14^ Without the confidence interval, the RIs for RBC‐I, MPV, HCT, RDW‐SD, and RDW‐CV were not verified. Classification of the XN‐V and XT‐2000iV results, using the Sysmex XT‐2000iV RIs, revealed very few discrepancies for most variables (Table S3).

## 4 DISCUSSION

According to the results obtained for the validation process based on the ASVCP and ICSH recommendations,^4^, ^6^ the Sysmex XN‐V hematology analyzer can be used for canine blood specimens.

The ASVCP guidelines help clinical pathologists assess the quality of analyzers.^3^ A first step can be to compare TE_obs_ with the TE_a_ proposed by experts for some variables.^15^, ^16^ In this study, all TE_obs_ values were lower than TE_a_ values, except for lymphocytes at low concentrations and monocytes within RIs. This was due to lymphocyte count imprecision and monocyte bias, according to the QGI calculation.^15^ The WBC differential count showed a higher TE_obs_ than the other hematology measurands in agreement with the recently proposed TE_a_.^15^, ^16^ Nevertheless, as recommended for all instruments, including the Sysmex XN‐V, the automated WBC differential of a patient can only be accepted after reviewing the scattergram and a blood smear to check automated findings.^16^

Carry‐over and linearity were excellent for all variables except lymphocytes. This was expected since similar results were obtained with the Sysmex XT‐2000iV analyzer.^7^, ^8^ However, in the previous studies, some variables, such as lymphocytes, were not studied. The imprecision of lymphocyte counting could explain why linearity was just intermediate and not excellent.

Comparison of the canine blood hematology results obtained with the Sysmex XT‐2000iV and Sysmex XN‐V analyzers was good to excellent for all the measured variables. These results were expected as the analyses, except for PLT‐F, involved the same analytical methods. Whereas the agreement of results between the Sysmex XN‐V and manual methods was good for HCT, neutrophil, and eosinophil counts, it was only fair for lymphocyte, monocyte, and NRBC counts, which was likely a consequence of the above‐mentioned imprecision.

A similar observation has already been reported for the Sysmex XT‐2000iV.^2^, ^17^ However, although the Sysmex XN‐V scattergrams were similar to those of the XT‐2000iV, they were not identical. The neutrophil and lymphocyte dot plots were more clearly separated on the Sysmex XN‐V. The main discrepancies between the manual and the Sysmex XN‐V WBC differentials were very similar to those described for the Sysmex XT‐2000iV (Figures 4 and 5).^2^, ^17^ In the case of high band cell concentrations and/or toxic neutrophil changes, the Sysmex XN‐V analyzer reported an incomplete or erroneous WBC differential count with or without a flag symbol beside the result or an error message. An increase in cytoplasmic RNA content shifted the band cells and/or toxic neutrophils upward in the DIFF scattergram, and these cells sometimes merged into the lymphocyte population, as previously described with the XT‐2000iV.^2^, ^17^ In acute leukemia or leukemic lymphoma, an absence of clear separation between the cellular lineages and possible presence of blastic cells in the monocyte cluster location have been previously reported with the XT‐2000iV.^17^ In our study, one case of acute leukemia showed similar features on the WDF scattergram obtained with the Sysmex XN‐V (Figure 5A). The extension of the leukocyte cluster to the upper right on the WNR scattergram (Figure 5B) was similar to a previous observation with the XT‐2000iV in a leukemia case, where cells located in the upper portion of the dot plot were designated lysis resistant cells.^18^ The high‐fluorescence clouds making up the reticulocyte population on the RET scattergram (Figure 5C) were suspected to be leukemic cells, as previously reported with the XT‐2000iV.^19^, ^20^ This clearer separation of the different leukocyte sub‐populations by the Sysmex XN‐V scattergrams should be used in the future by the manufacturer to improve the precision of counts and thus reduce the number of flags and error messages. Even though the correlation for NRBC was only fair, the slope and intercept of the Passing‐Bablok regression equation were close to 1 and equal to 0, respectively. The low correlation could have resulted from very few NRBCs and the poor reliability of manual NRBC counting.

Our study revealed significant effects of hemoglobinemia and lipemia on HGB and associated variables. Furthermore, lipemia and bilirubinemia also had a significant effect on PLT counts. ASVCP recommendations^3^, ^21^ for instrument validation include interference studies, but a search in PubMed for interference studies with hematology analyzers indicated that no publications are available. This is quite surprising, as hemolysis and hyperlipemia are known to artifactually increase HGB measurements using spectrophotometry.^22^ Publications on erroneous cell counts or HGB measurements are more numerous than those on hemolysis, and it is interesting to note that interfering substances can cause different types of errors depending on the analyzer. For laser‐based hematology analyzers, such as the ADVIA 120 or 2120, which measure the hemoglobin concentration within each red blood cell and generate so‐called measured MCHC or CHCM, a difference between the CHCM and the calculated MCHC is observed with an artifactual increase of MCHC but not of CHCM.^22^, ^23^ It was interesting to note that, with the Sysmex XN‐V, no effects of hemoglobin, lipemia, or bilirubin on RET‐He or RBC‐He (which were also measured using a laser beam) were observed.

The mean RBC variable values after storage for up to 72 hours were significantly different from *T*_0_. Such results are well‐known and secondary to red blood cell swelling, as previously reported in numerous studies.^8^, ^17^, ^24^, ^25^, ^26^, ^27^, ^28^, ^29^ By contrast, stability is not often reported in validation studies,^17^ probably because it is not included in the ASVCP recommendations. However, in the ICSH guidelines for evaluating blood cell analyzers in human medicine, specimen stability testing at room temperature and 4℃ after 4, 8, 12, 24, 48, and 72 hours of storage is recommended.^6^ Three studies have reported stability of canine blood with the Sysmex XT‐2000iV,^8^, ^17^, ^25^ yielding similar results to those of the Sysmex XN‐V, with a significant increase in the HCT, MCV, and RET, and a decrease in the MCHC, monocytes, and PLT; the reported changes were more intense at 4℃ than at room temperature, except for PLT.^17^, ^25^

According to the IFCC, CLSI, and ASVCP guidelines, validation of a pre‐existing RI can be accepted if one of the following three procedures is employed: subjective assessment, validation using small numbers of reference individuals, or validation using large numbers of reference individuals.^11^, ^12^, ^13^ In our study, we chose the second option, in which acceptability is based on determining reference values in 20 reference individuals and comparing these reference values to the RIs of a larger original study.^14^ The CLSI and ASVCP acceptance criterion is: “If ≤2 of the 20 values fall outside the candidate RI, it is considered transferable.” This requirement was met for most variables. These RIs can, therefore, be used to interpret results obtained with the Sysmex XN‐V for the validated variables, pending de novo determination of RIs for new variables, such as PLT‐F, for RIs that have not been established, such as RET‐He, and RBC‐He, and finally for RIs where acceptability was not attained in our study, such as RDW or MPV.

To conclude, the Sysmex XN‐V results can readily be trusted for canine blood hematology analyses, even if the lymphocyte and monocyte concentrations need to be confirmed with a blood smear examination. Lipemia, hemolysis, scattergrams, and flags or error messages should be checked before validating results. Finally, specimens should be analyzed as early as possible, a 24‐hour delay being acceptable if the specimen is stored at 4℃, and similar species‐specific studies are necessary for valid use of the XN analyzer in other species.

## CONFLICT OF INTEREST

The authors do not have any conflict of interest to disclose.

## DISCLOSURE

The authors have indicated that they have no affiliations or financial involvement with any organization or entity with a financial interest in, or in financial competition with, the subject matter or materials discussed in this article.

## Supporting information

Fig S1

Fig S2

Fig S3

Fig S4

Table S1‐4

Supplementary Material
