# Supplementary figures and images for: Validation of the Sysmex XN‐V hematology analyzer for canine specimens
Source: Vet Clin Pathol. 2021 Jun 21;50(2):184–97. doi: 10.1111/vcp.12936 (PMC8362000; doi:10.1111/vcp.12936)

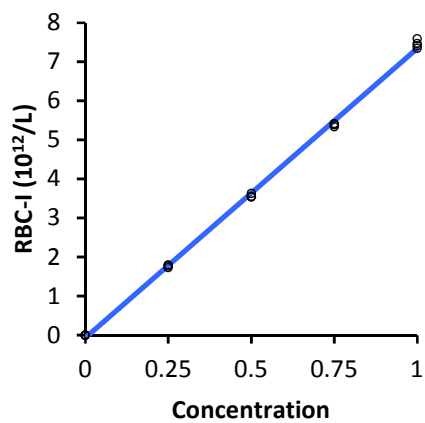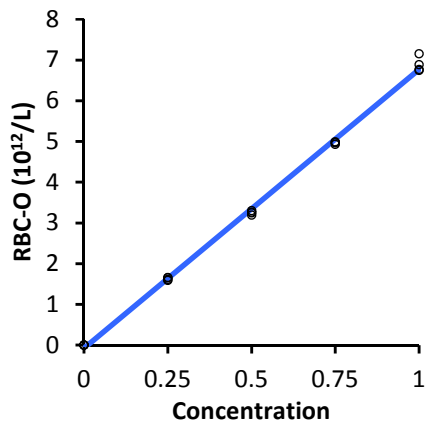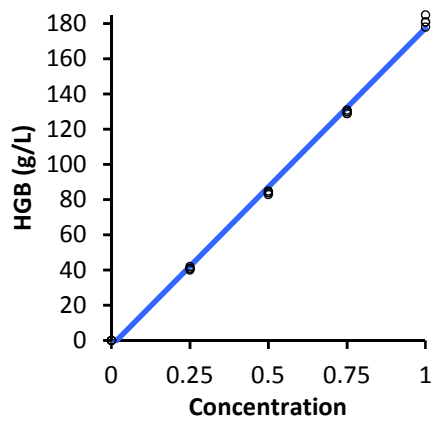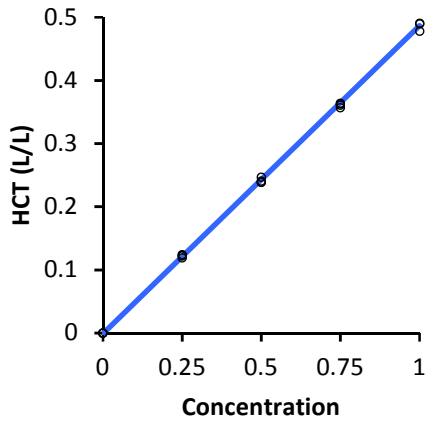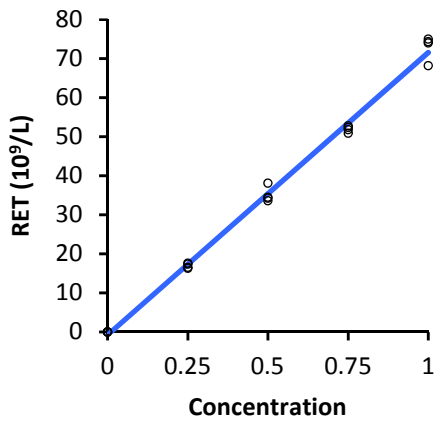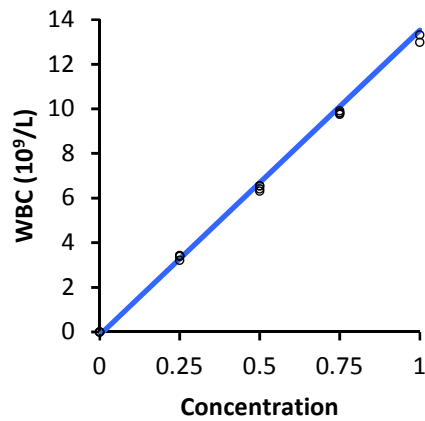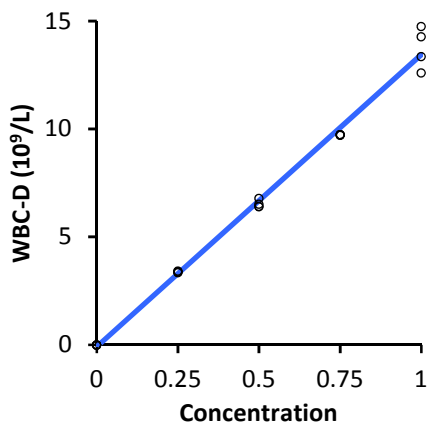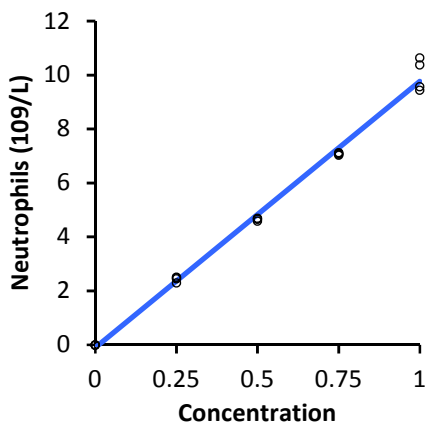

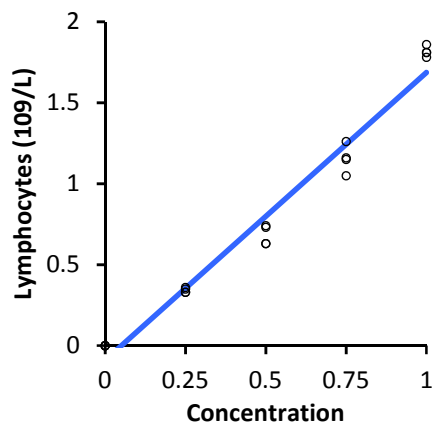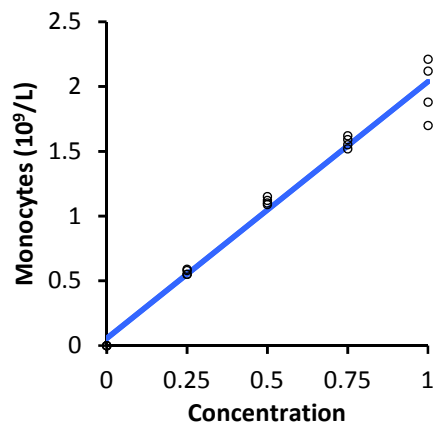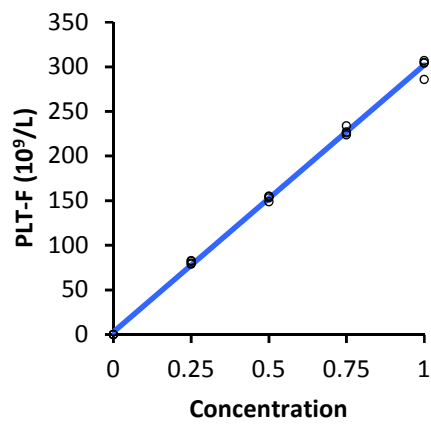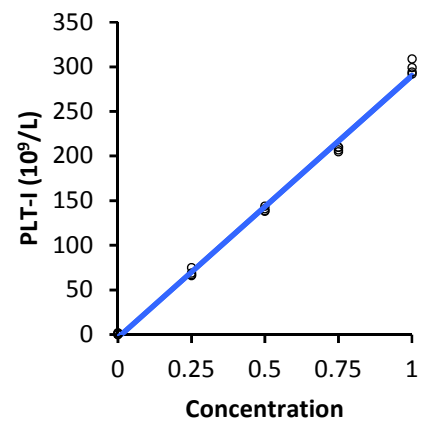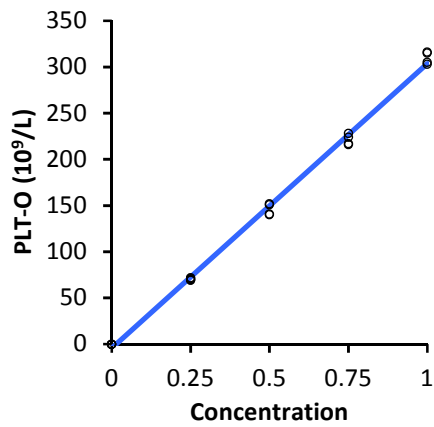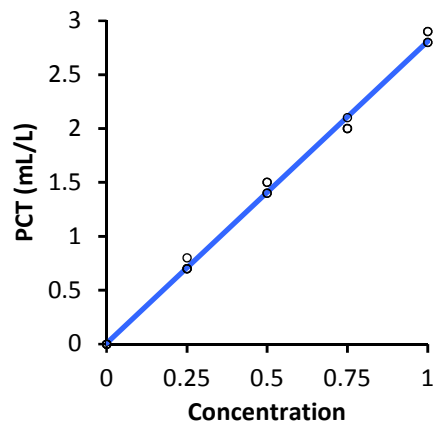

Supplement: Supplementary file 1 — Fig S1 [file VCP-50-184-s004.pdf]

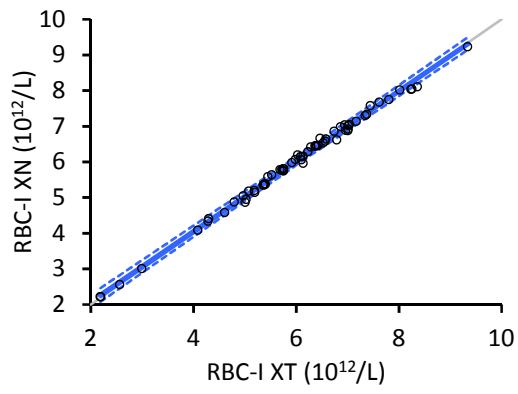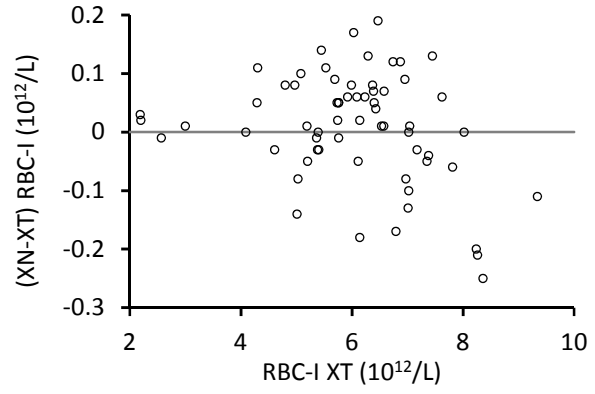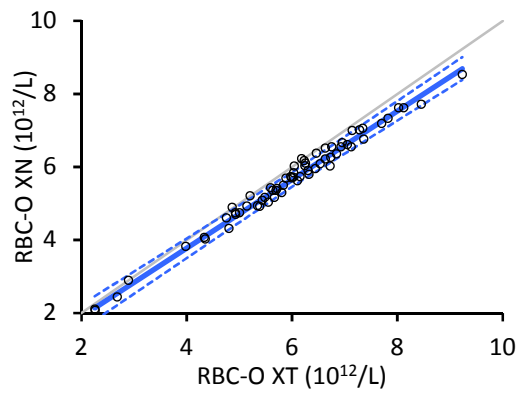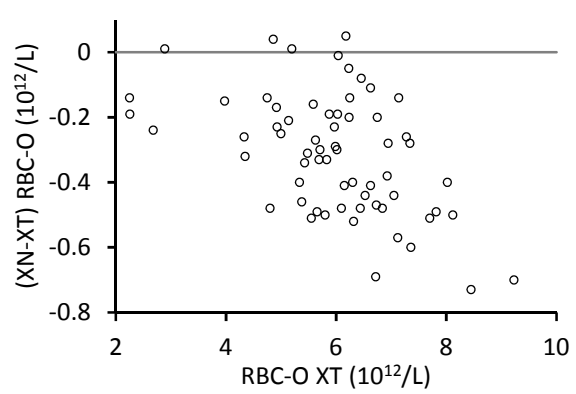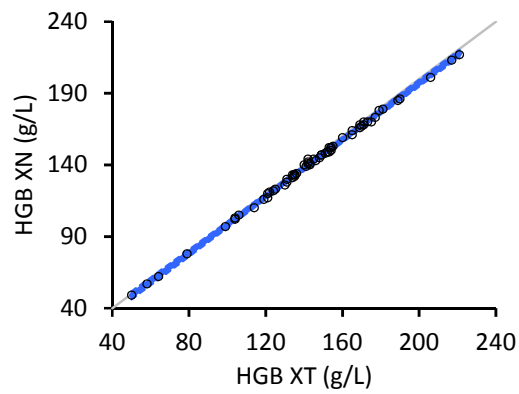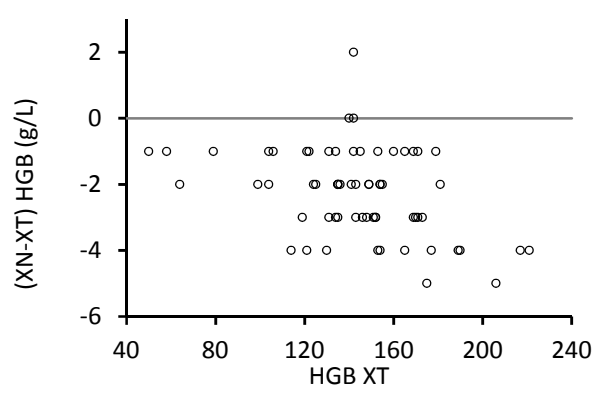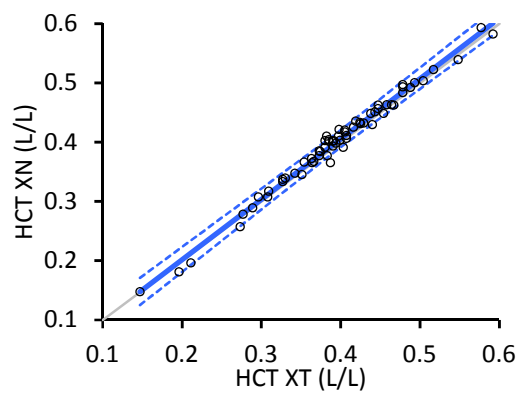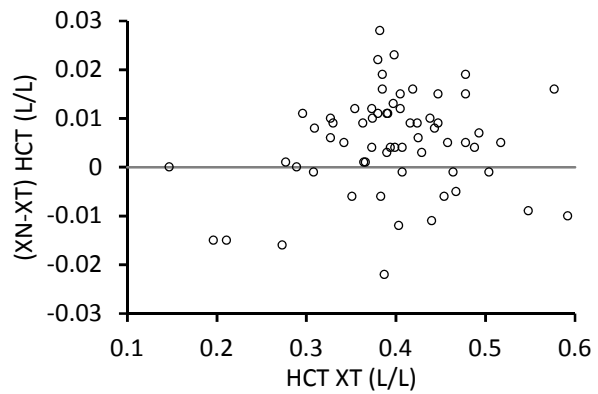

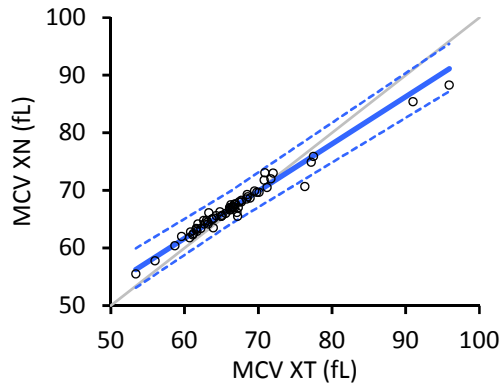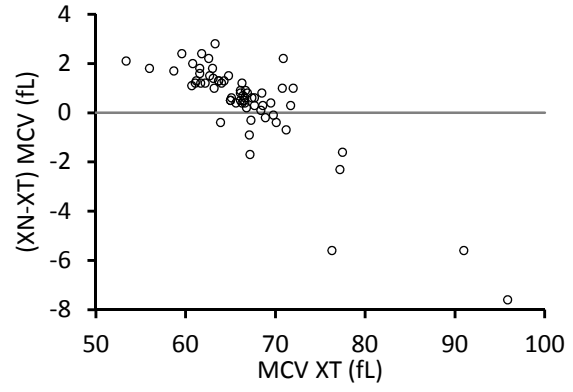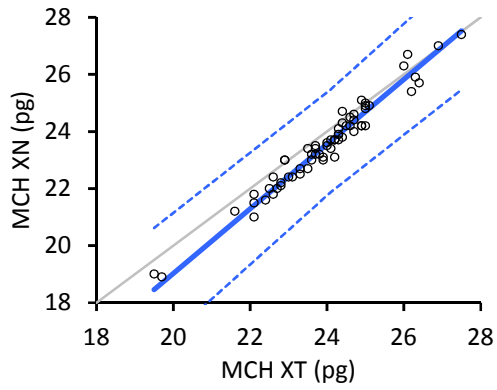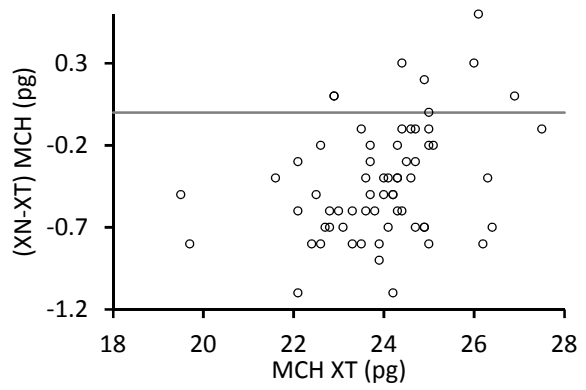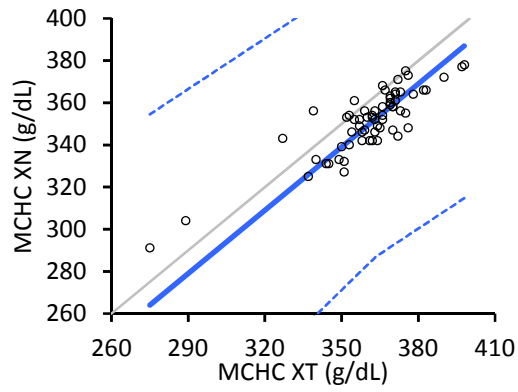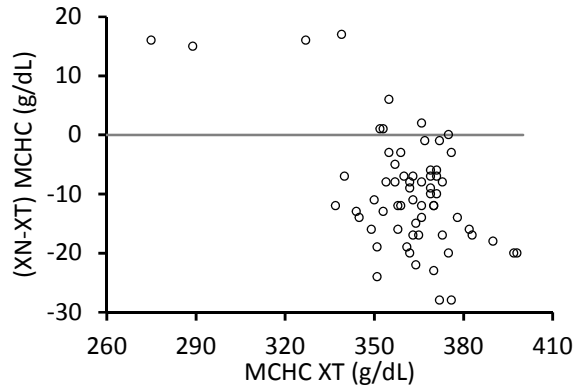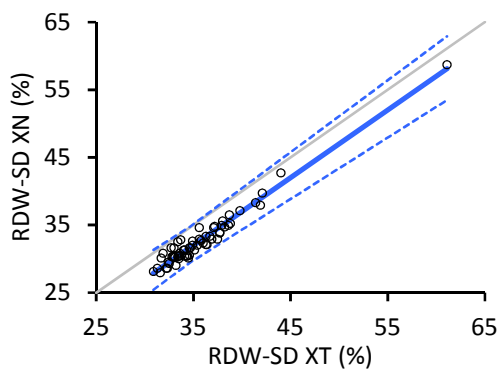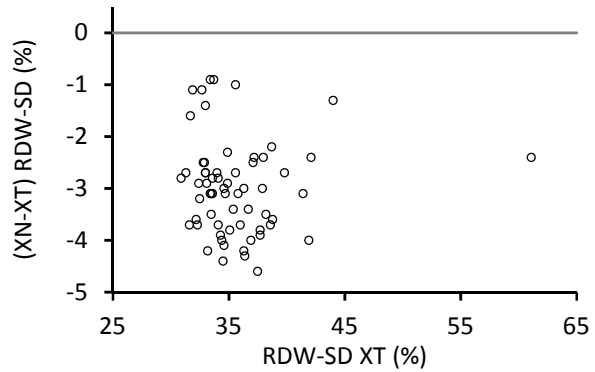

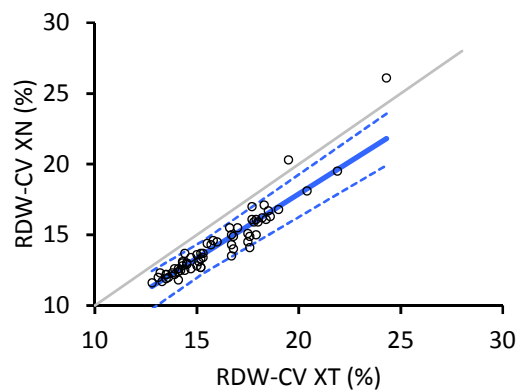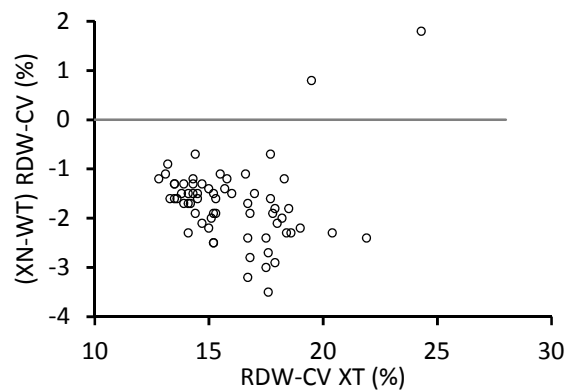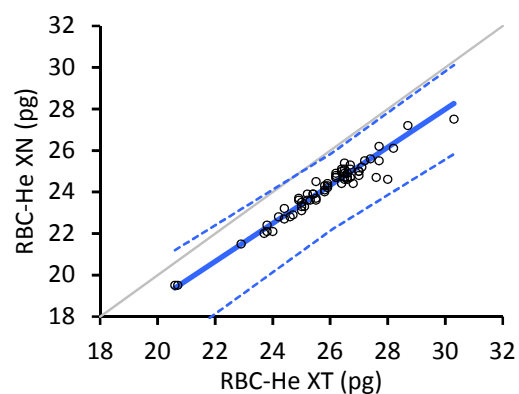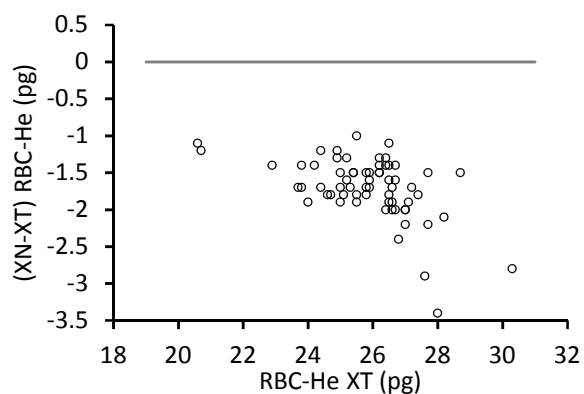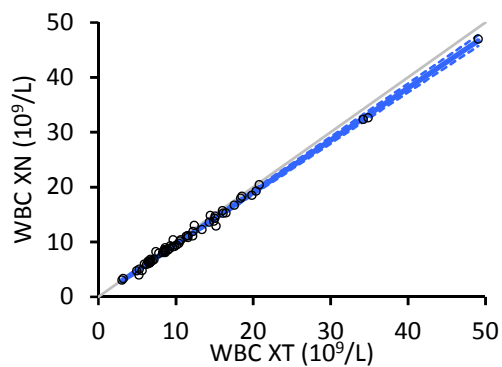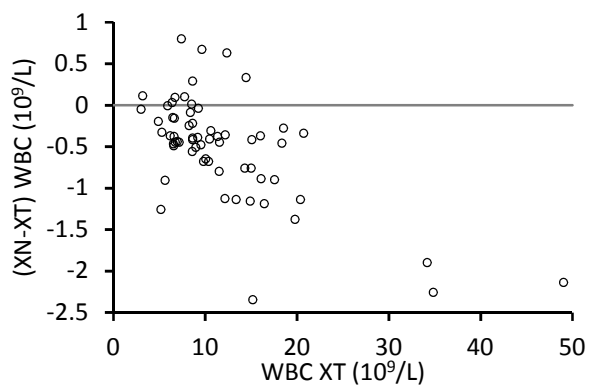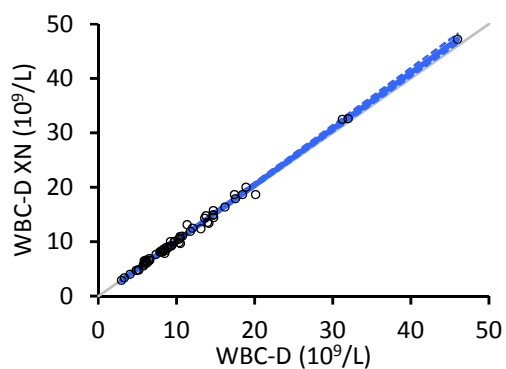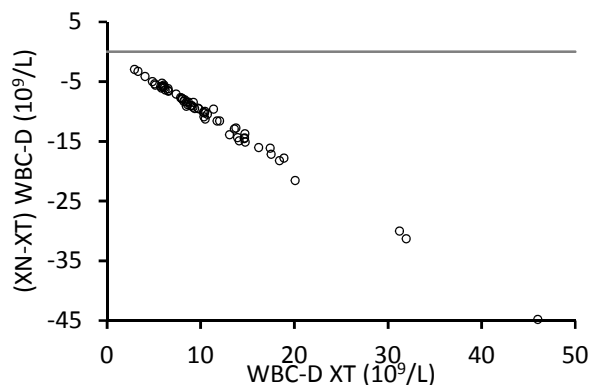

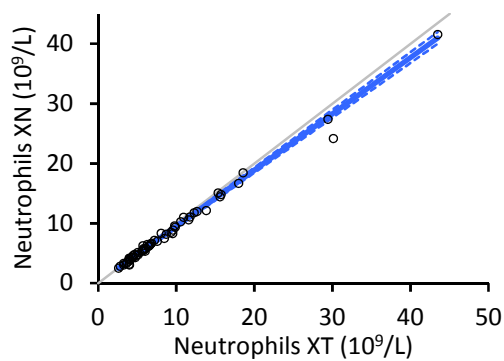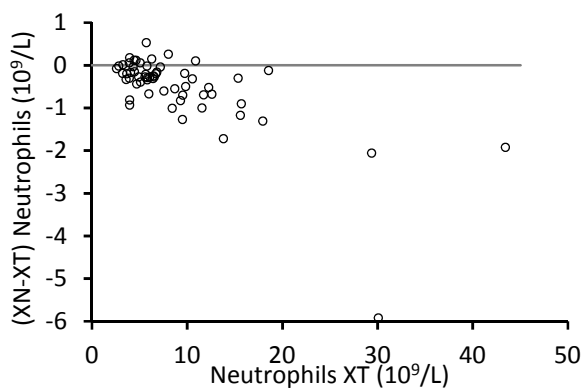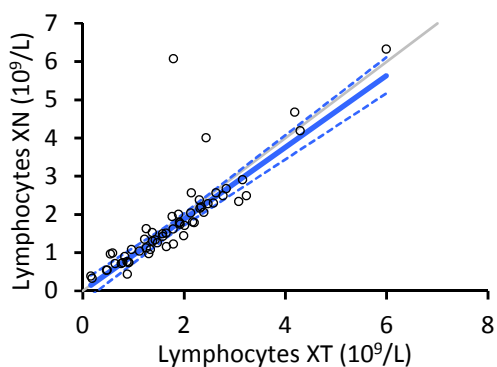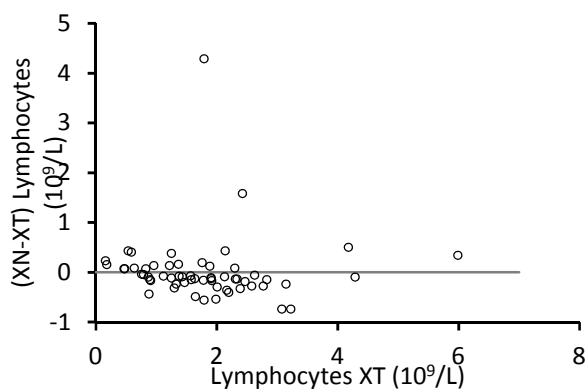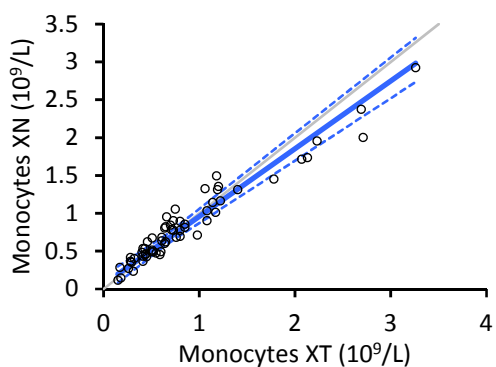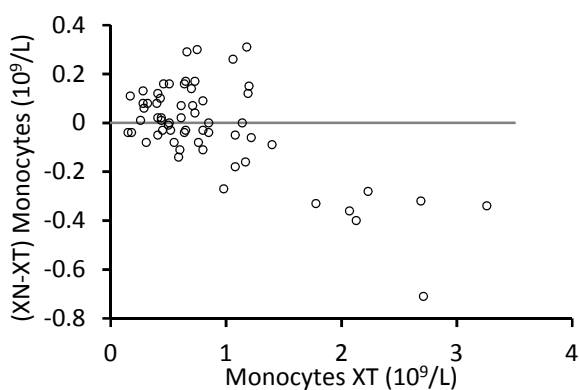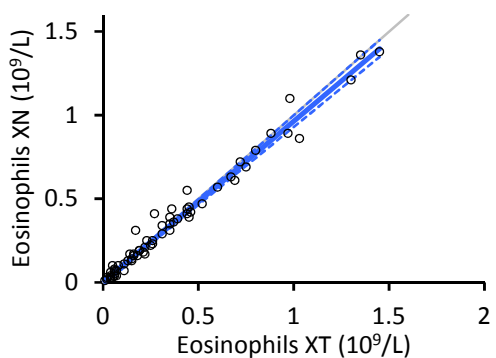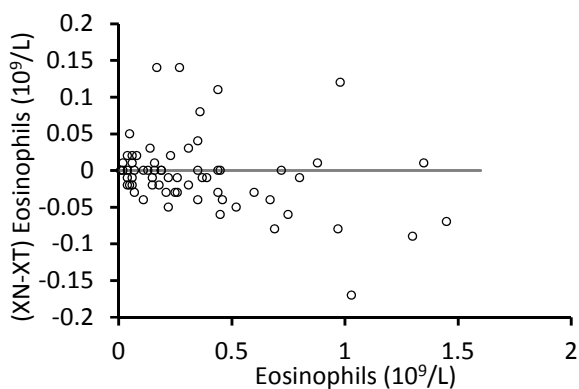

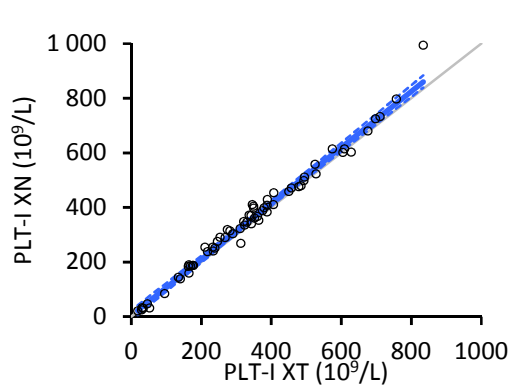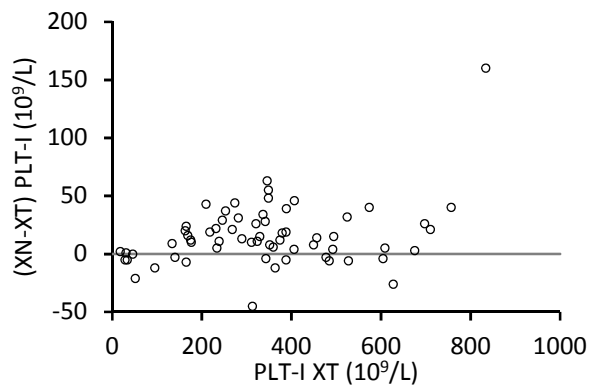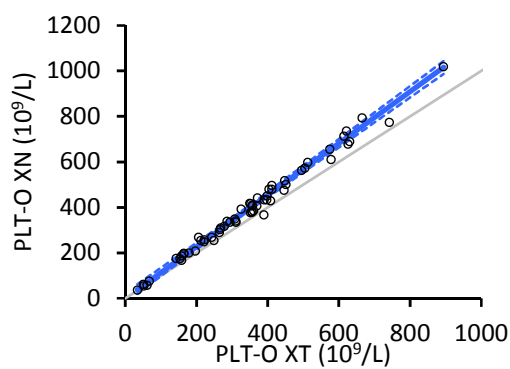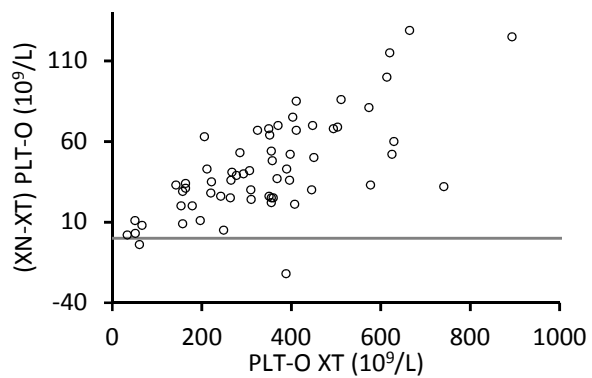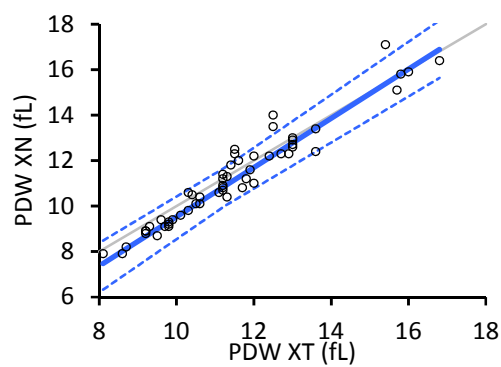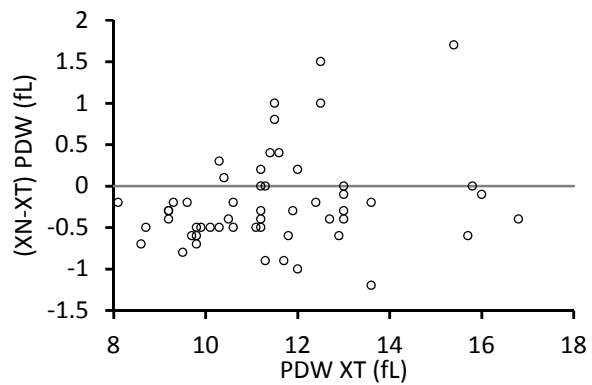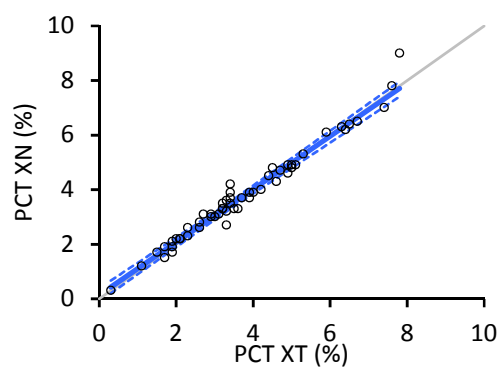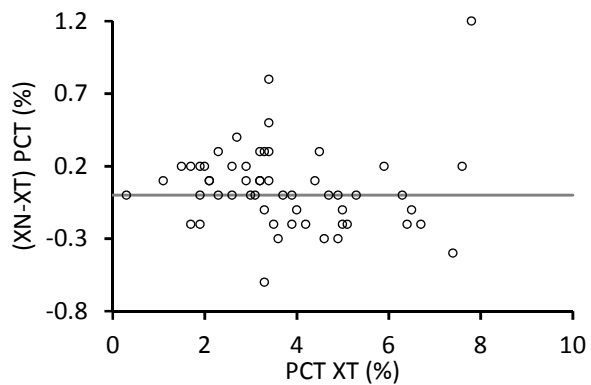

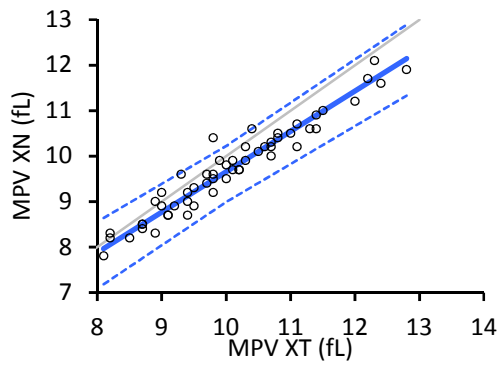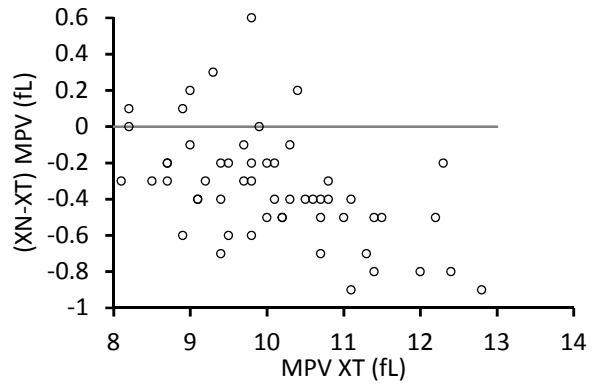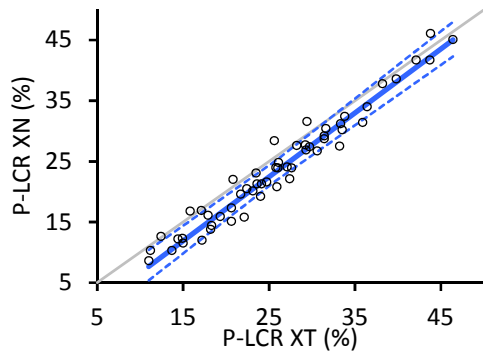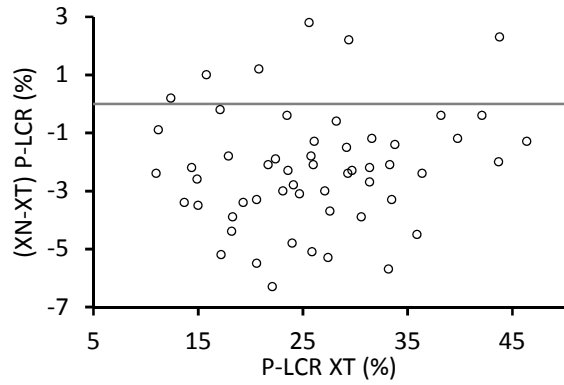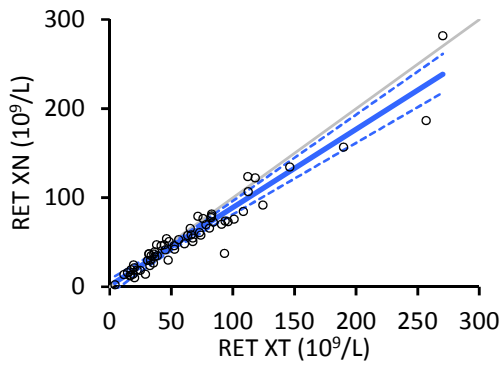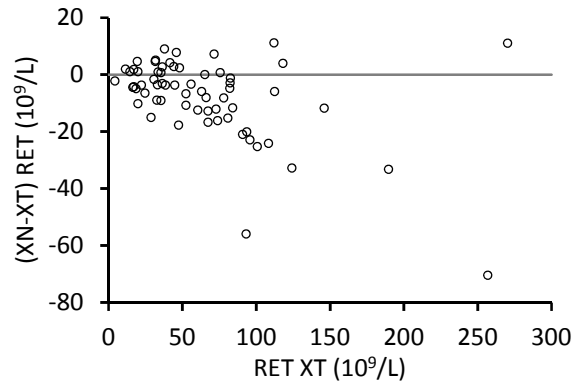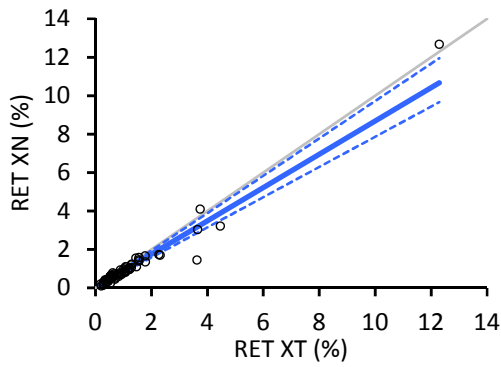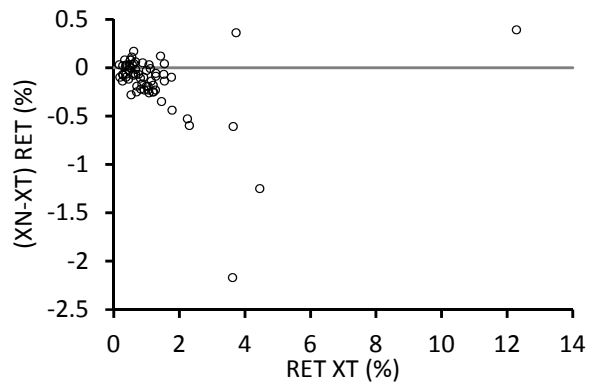

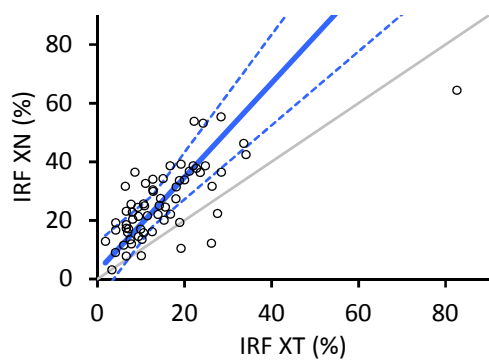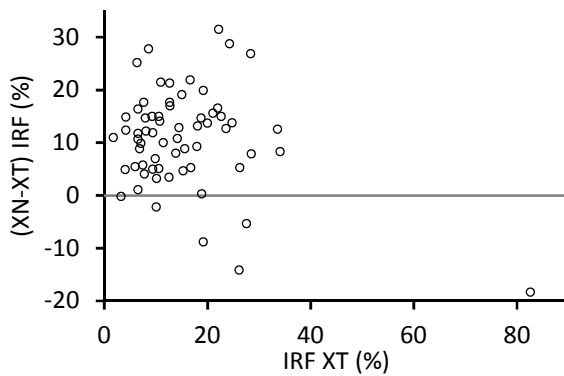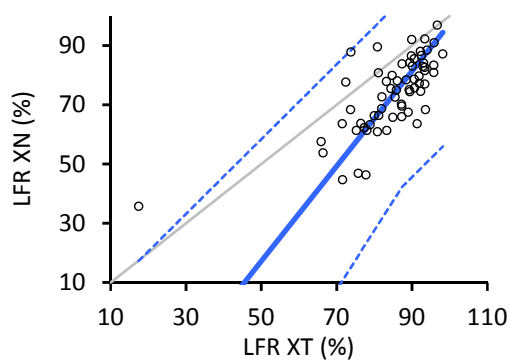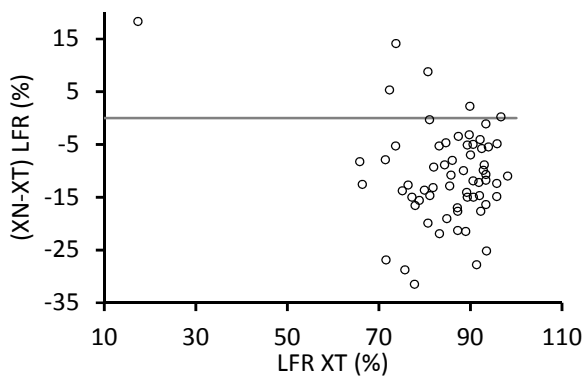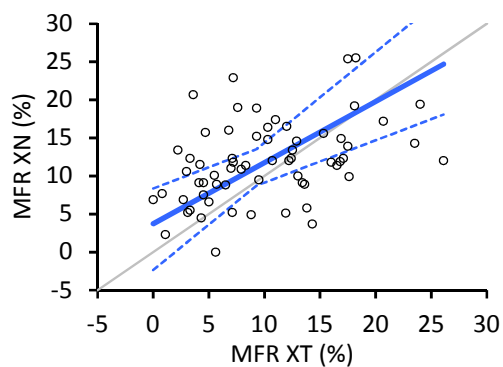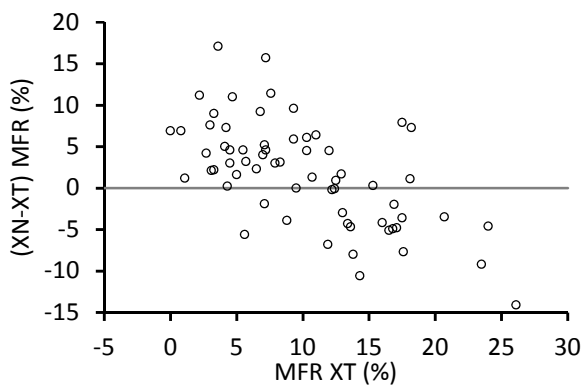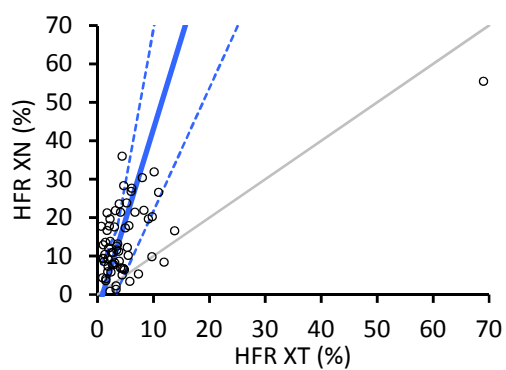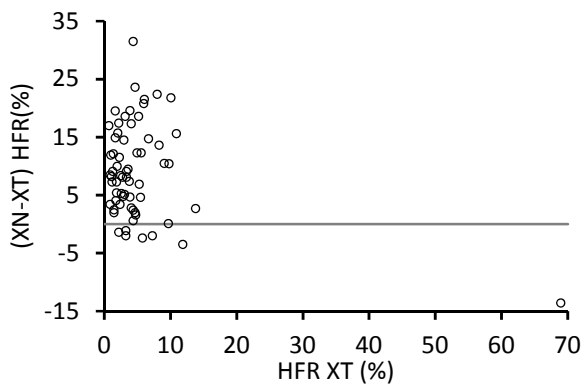

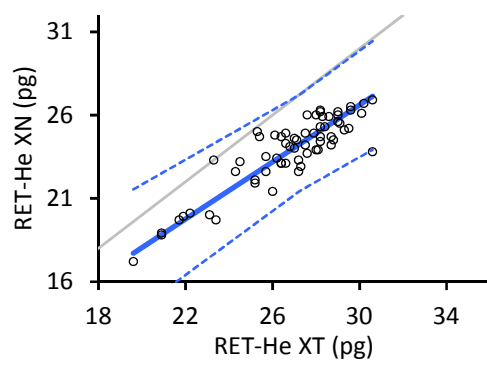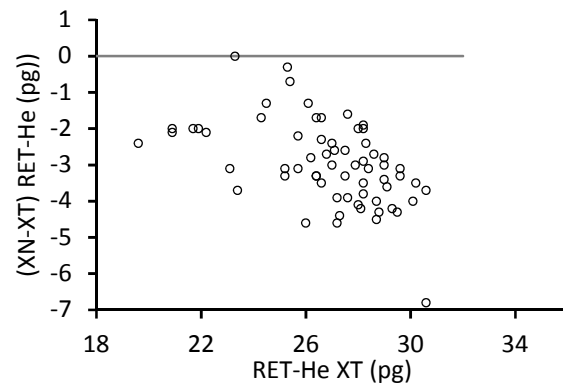

Supplement: Supplementary file 2 — Fig S2 [file VCP-50-184-s002.pdf]

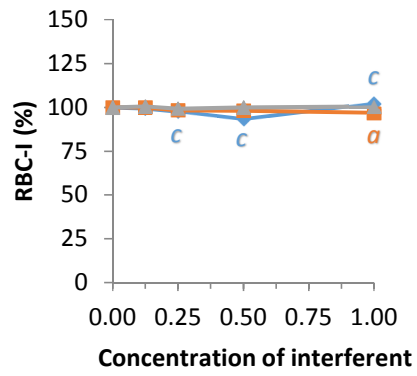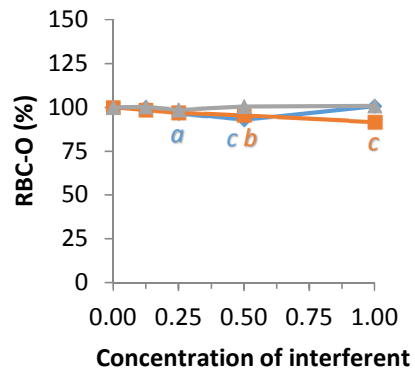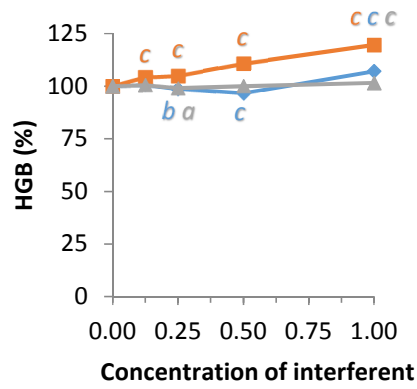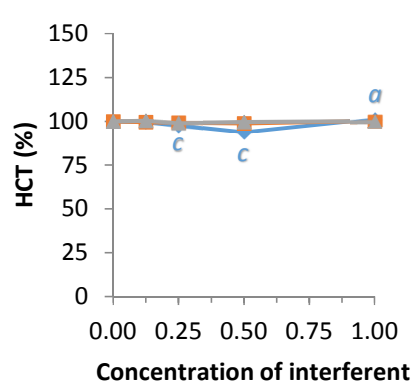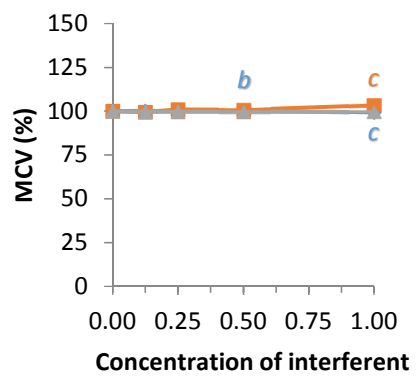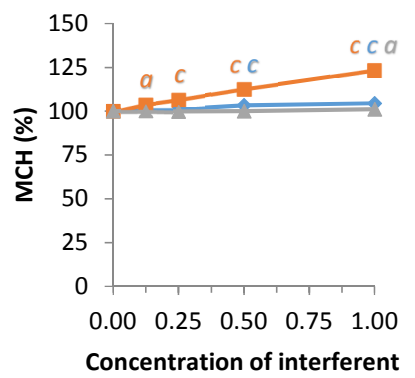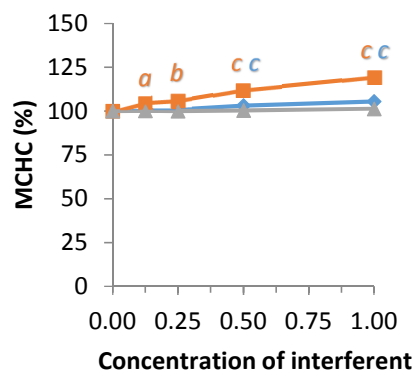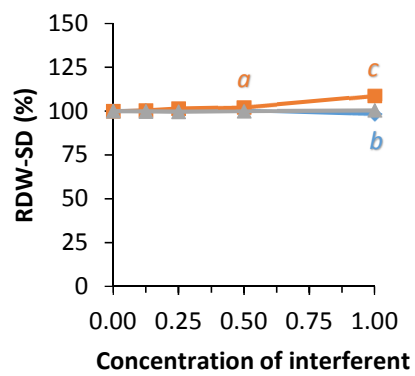

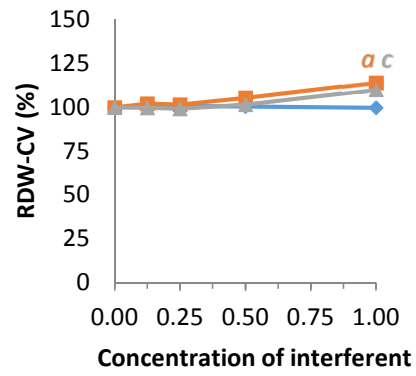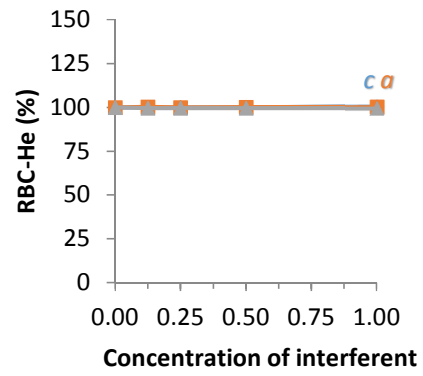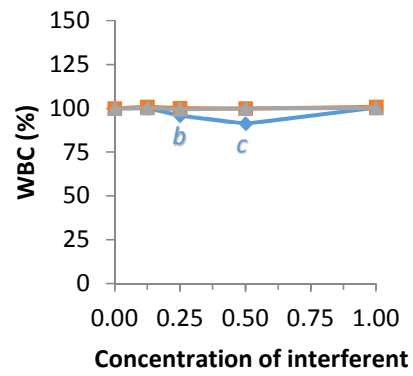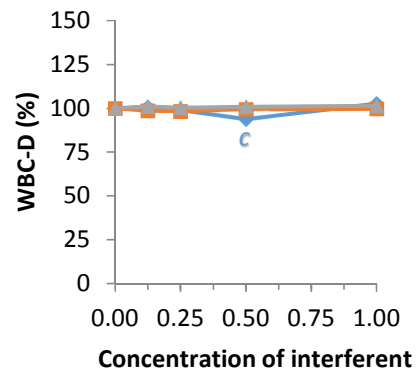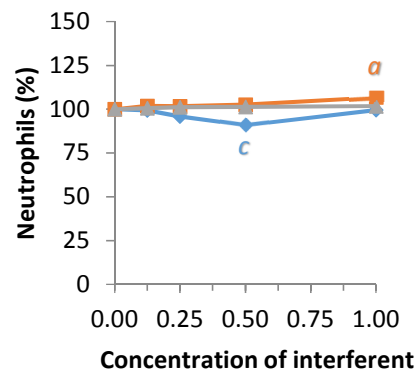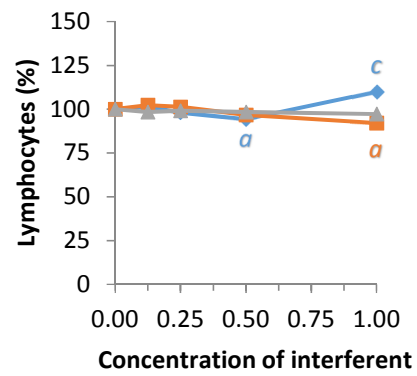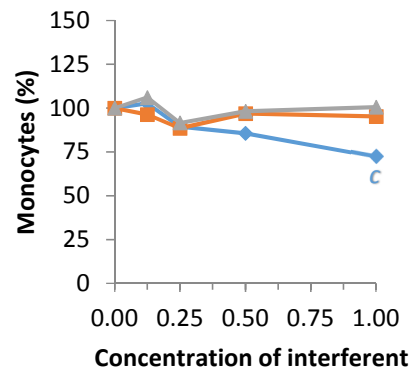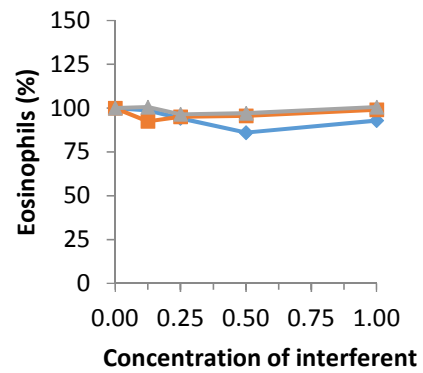

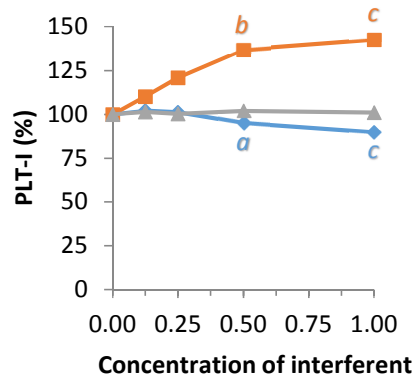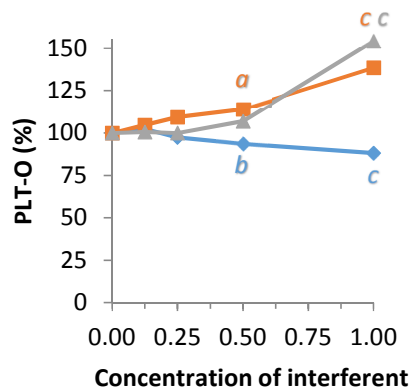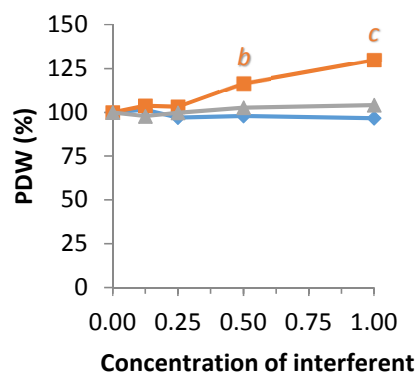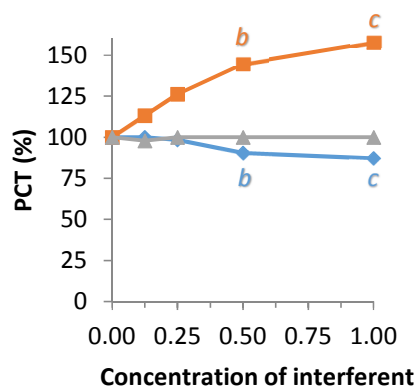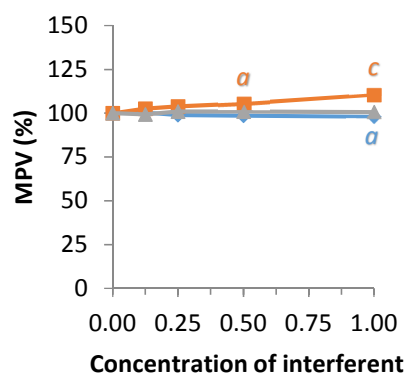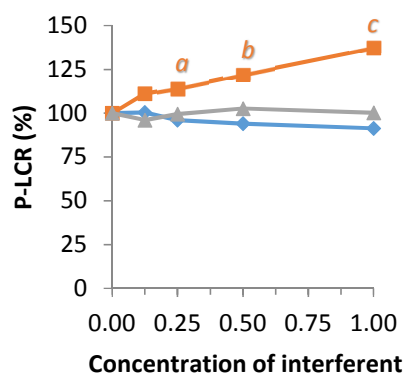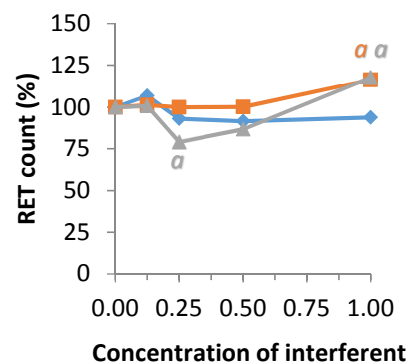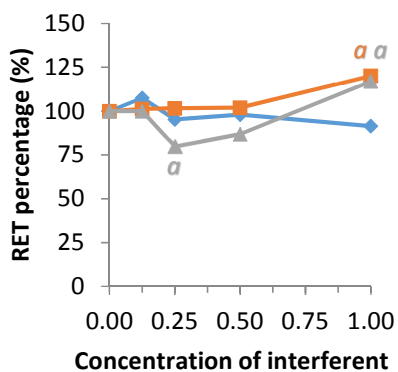

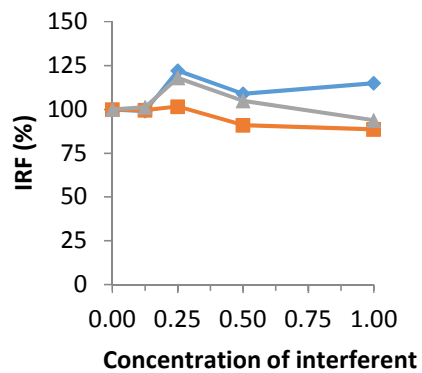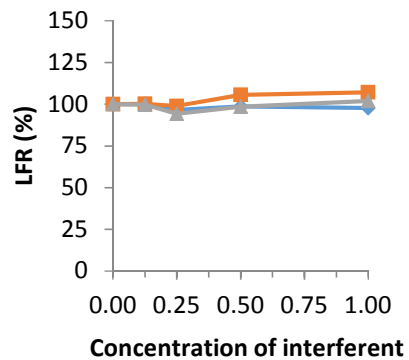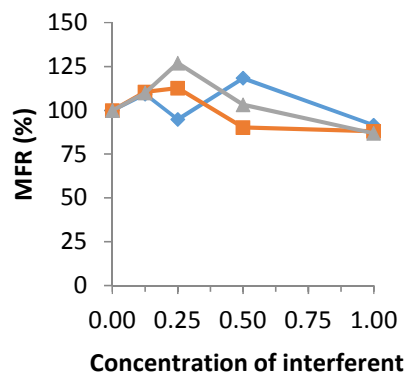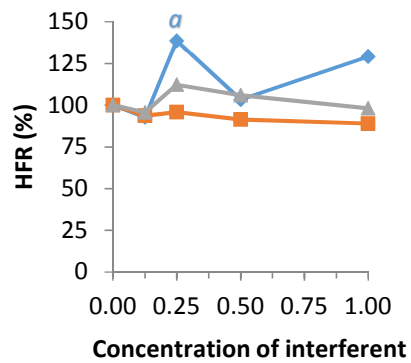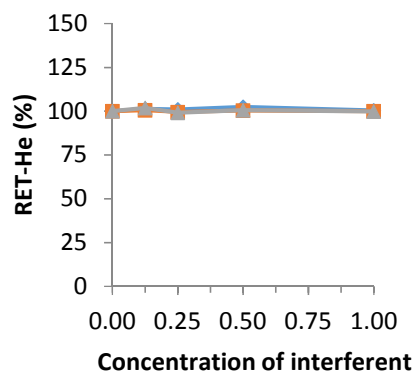

Supplement: Supplementary file 3 — Fig S3 [file VCP-50-184-s001.pdf]

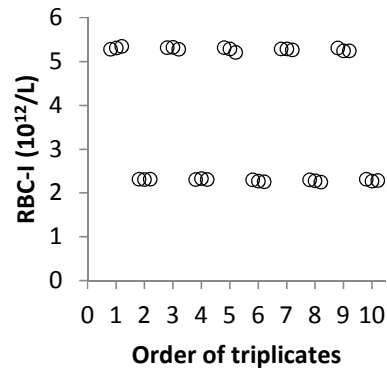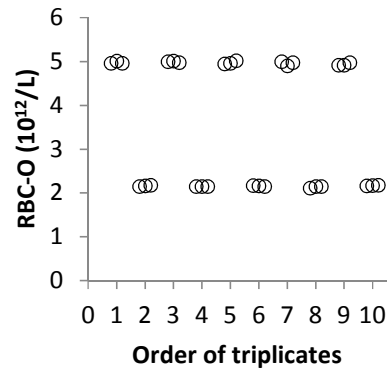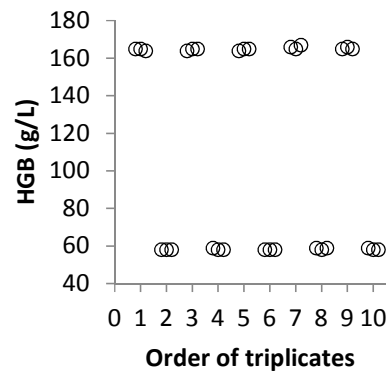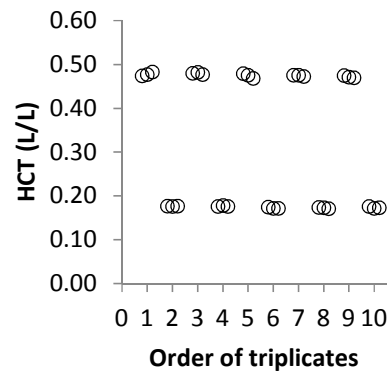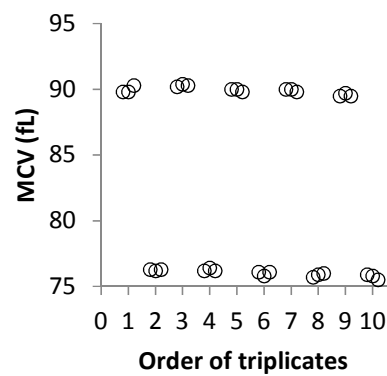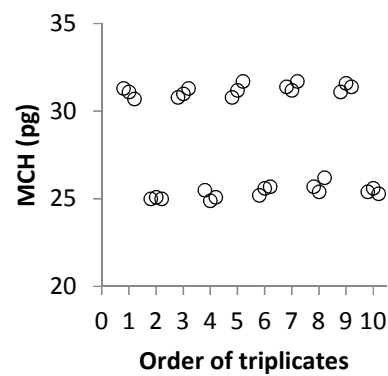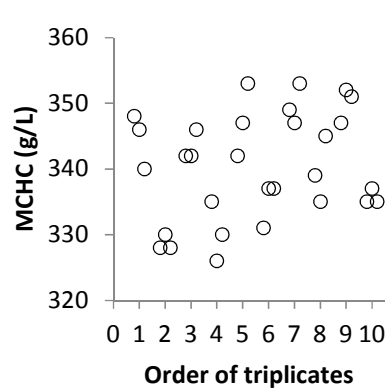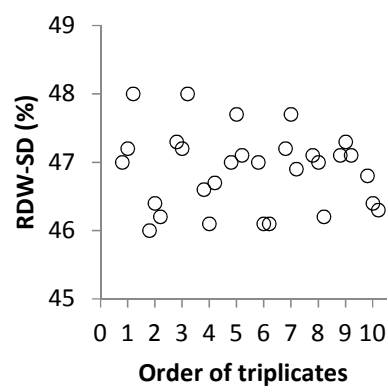

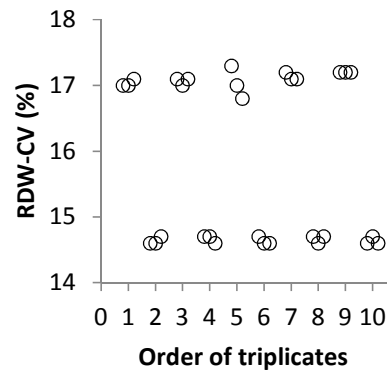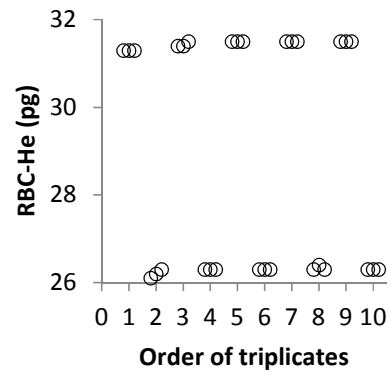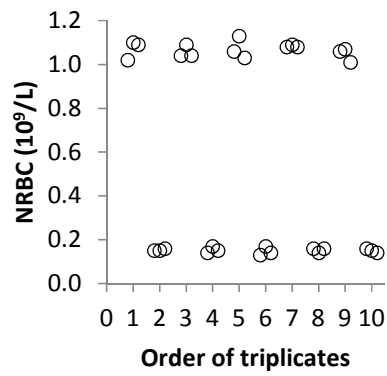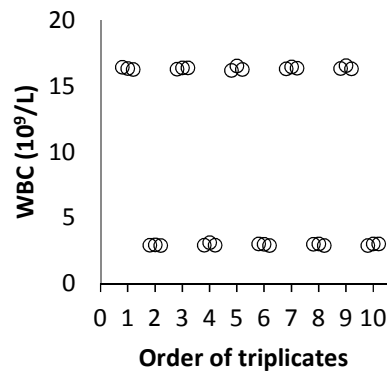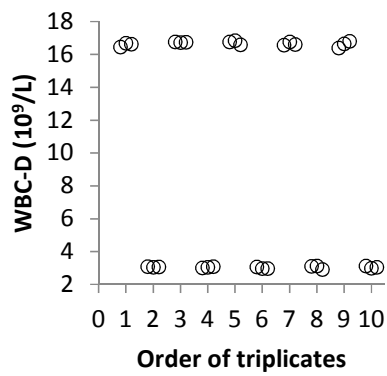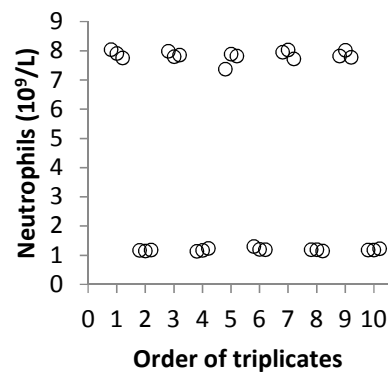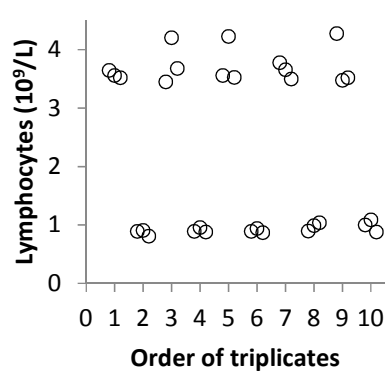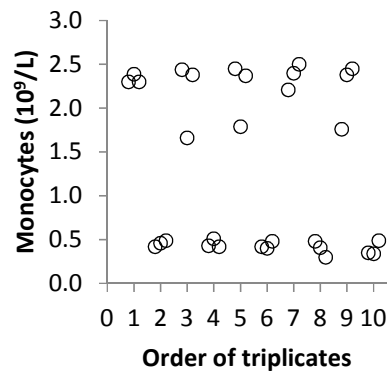

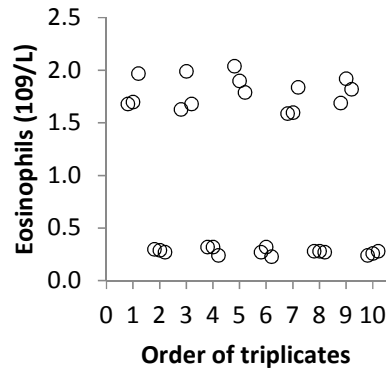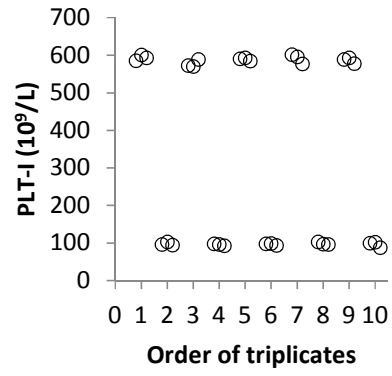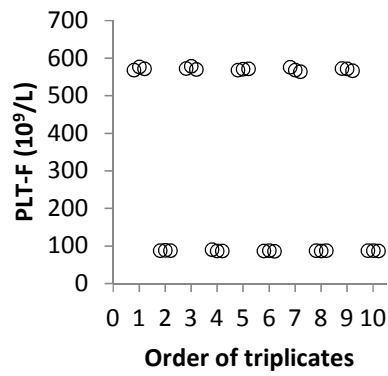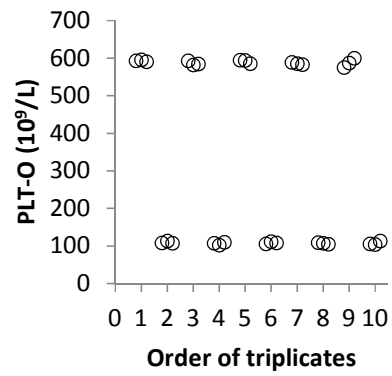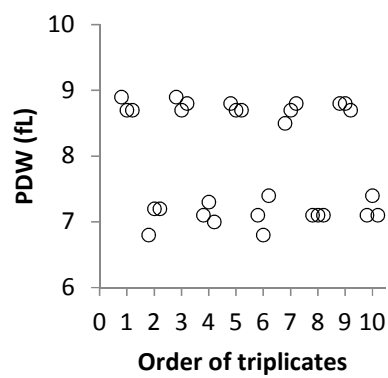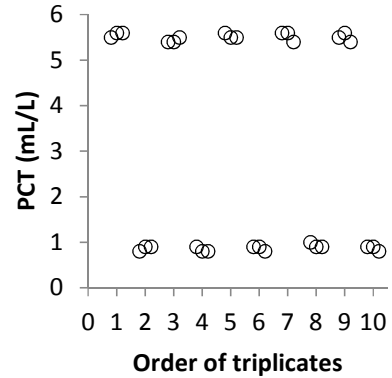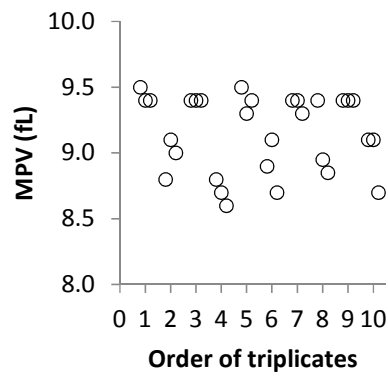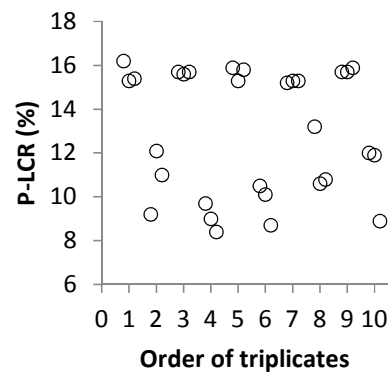

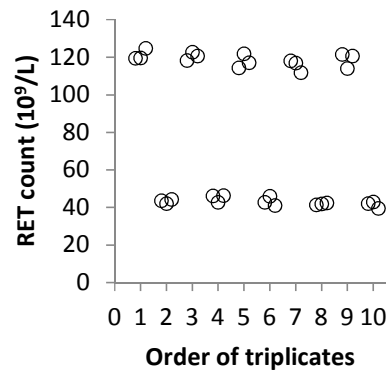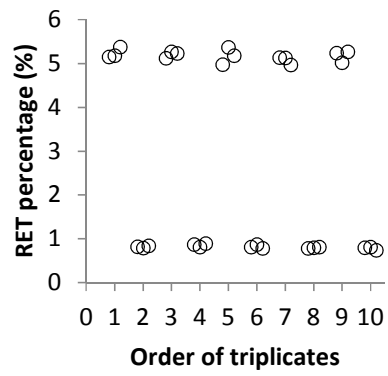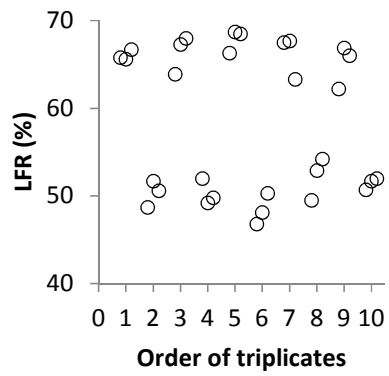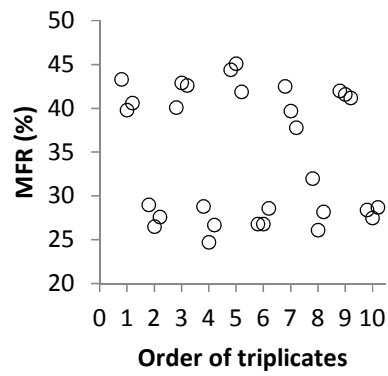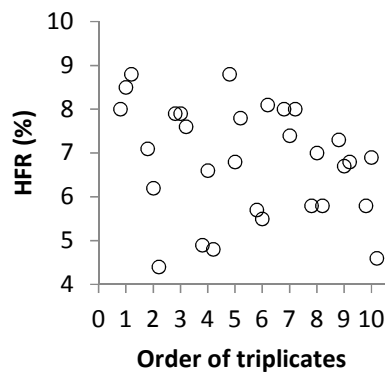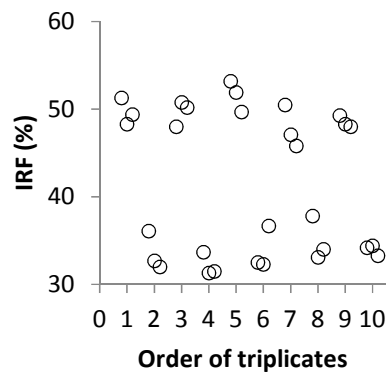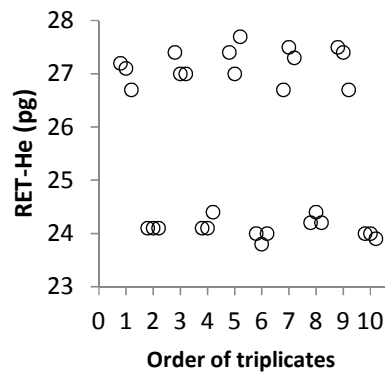

Supplement: Supplementary file 4 — Fig S4 [file VCP-50-184-s003.pdf]
