# Supplementary material for: Validation of the Sysmex XN‐V hematology analyzer for canine specimens
Source: Vet Clin Pathol. 2021 Jun 21;50(2):184–97. doi: 10.1111/vcp.12936 (PMC8362000; doi:10.1111/vcp.12936)
Supplement: Supplementary file 5 — Table S1‐4 [file VCP-50-184-s006.docx]

**Table S1.** Imprecision, bias and total error for the Sysmex XN-V variables with quality control solutions (QC1 and QC2) and a randomly selected canine specimen.

| Variable | Unit | QC1 solution | | | | | | QC2 solution | | | | | | | Canine Blood | |  | QGI |
| --- | --- | --- | --- | --- | --- | --- | --- | --- | --- | --- | --- | --- | --- | --- | --- | --- | --- | --- |
|  |  | Target | Repeat | Reprod | Bias  % | TE_obs_^*^  % |  | | Target | Repeat | Reprod | Bias  % | TE_obs_^*^  % |  | Mean  value | Repeat  CV % | TE_a_^*^  % |  |
|  |  |  | CV % | |  |  |  |  |  | CV % | |  |  |  |  |  |  |  |
| RBC-I | 10^12^/L | 2.32 | 0.55 | 1.07 | 1.88 | 4.02 |  | | 4.36 | 0.55 | 0.83 | 1.23 | 2.89 |  | 5.59 | 1.08 | 10 |  |
| RBC-O | 10^12^/L | 2.31 | 1.73 | 1.97 | -3.81 | 7.75 |  | | 4.22 | 1.78 | 1.84 | -2.93 | 6.61 |  | 5.20 | 0.72 |  |  |
| HGB | g/L | 59 | 1.07 | 1.26 | 1.69 | 4.21 |  | | 126 | 0.70 | 0.70 | 0.48 | 1.88 |  | 146 | 0.75 | 10 |  |
| HCT | L/L | 0.175 | 0.64 | 1.91 | 3.00 | 6.82 |  | | 0.367 | 0.54 | 1.49 | 2.62 | 5.60 |  | 0.396 | 0.64 | 10 |  |
| MCV | fL | 75.3 | 0.05 | 0.12 | 1.27 | 1.51 |  | | 84.1 | 0.16 | 0.78 | 1.45 | 3.01 |  | 70.9 | 1.43 | 7 |  |
| MCH | pg | 25.4 | 0.09 | 0.12 | -0.10 | 0.34 |  | | 28.9 | 1.02 | 1.02 | -0.71 | 2.75 |  | 26.2 | 0.68 |  |  |
| MCHC | g/L | 337 | 1.00 | 1.82 | -1.22 | 4.86 |  | | 343 | 0.89 | 1.52 | -1.98 | 5.02 |  | 369 | 1.21 | 10 |  |
| RDW-SD | fL | 45.0 | 0.85 | 0.99 | 1.24 | 3.22 |  | | 45.7 | 0.54 | 0.93 | 3.02 | 4.88 |  | 31.1 | 1.73 |  |  |
| RDW-CV | % | 16.8 | 0.68 | 0.85 | -0.36 | 2.06 |  | | 15.5 | 0.04 | 0.06 | -0.42 | 0.54 |  | 11.8 | 0.48 |  |  |
| RBC-He | pg | 26.6 | 0.19 | 0.45 | 0.21 | 1.11 |  | | 29.4 | 0.17 | 0.29 | 1.34 | 1.92 |  | 26.6 | 0.28 |  |  |
|  |  |  |  |  |  |  |  | |  |  |  |  |  |  |  |  |  |  |
| WBC | 10^9^/L | 3.06 | 1.54 | 2.00 | -0.21 | 4.21 |  | | 6.86 | 0.72 | 1.05 | -1.46 | 3.56 |  | 7.59 | 1.36 | 15 |  |
| WBC-D | 10^9^/L | 3.10 | 1.57 | 1.68 | -0.15 | 3.51 |  | | 7.02 | 1.60 | 1.60 | -1.33 | 4.53 |  | 7.61 | 1.14 |  |  |
| Neutrophils | 10^9^/L | 1.16 | 4.46 | 4.53 | 3.28 | 12.34 |  | | 2.97 | 2.89 | 3.15 | -0.59 | 6.89 |  | 4.08 | 1.87 | 15 |  |
| Lymphocytes | 10^9^/L | 0.97 | 8.43 | 8.43 | -3.56 | 20.42 |  | | 1.82 | 2.45 | 3.22 | -4.70 | 11.14 |  | 2.55 | 2.40 | 15 | 0.28 |
| Monocytes | 10^9^/L | 0.50 | 15.66 | 17.21 | -0.20 | 34.62 |  | | 1.04 | 4.29 | 5.64 | 66.78 | 78.06 |  | 0.52 | 7.47 | 60^b^ & 50^c^ | 8.97 |
| Eosinophils | 10^9^/L | 0.28 | 11.12 | 11.32 | 0.00 | 22.64 |  | | 0.70 | 12.71 | 14.21 | -1.86 | 30.28 |  | 0.40 | 5.17 | 90^a^, 50^b^ & 50^c^ |  |
|  |  |  |  |  |  |  |  | |  |  |  |  |  |  |  |  |  |  |
| PLT-I | 10^9^/L | 91 | 3.00 | 7.70 | 4.89 | 20.29 |  | | 243 | 1.67 | 2.42 | 6.85 | 11.7 |  | 201 | 2.93 | 20 |  |
| PLT-F | 10^9^/L | 85 | 3.05 | 3.45 | 1.18 | 8.08 |  | | 255 | 0.96 | 2.11 | 10.45 | 14.67 |  | 203 | 1.74 | 20 |  |
| PLT-O | 10^9^/L | 98 | 4.93 | 6.44 | 11.68 | 24.56 |  | | 252 | 2.55 | 3.45 | 9.58 | 16.48 |  | 207 | 3.17 | 20 |  |
| PDW | fL | 8.9 | 2.62 | 2.89 | 26.80 | 32.60 |  | | 8.5 | 2.67 | 2.78 | 8.29 | 13.85 |  | 11.3 | 3.38 |  |  |
| PCT | mL/L | 0.7 | 7.14 | 11.90 | 15.00 | 38.8 |  | | 2.4 | 2.80 | 3.13 | 6.88 | 13.14 |  | 2.0 | 2.96 |  |  |
| MPV | fL | 8.0 | 2.92 | 3.33 | 6.44 | 13.10 |  | | 9.8 | 1.19 | 1.25 | 1.07 | 3.57 |  | 9.9 | 0.75 |  |  |
| P-LCR | % | 4.8 | 36.28 | 41.50 | 81.46 | 164.46 |  | | 18.9 | 5.16 | 5.26 | 8.20 | 18.72 |  | 24.3 | 3.80 |  |  |
| IPF | % | 19.3 | 1.60 | 1.84 | 0.36 | 4.04 |  | | 19.9 | 3.46 | 4.08 | -0.58 | 8.74 |  | 1.0 | 8.58 |  |  |
|  |  |  |  |  |  |  |  | |  |  |  |  |  |  |  |  |  |  |
| RET | 10^9^/L | 134 | 2.13 | 2.55 | -4.27 | 9.37 |  | | 112 | 2.78 | 2.85 | -2.97 | 8.67 |  | 11.8 | 5.03 | 20 |  |
| RET | % | 5.78 | 2.25 | 2.46 | -6.03 | 10.95 |  | | 2.56 | 2.69 | 3.16 | -4.16 | 10.48 |  | 0.19 | 9.33 |  |  |
| IRF | % | 35.0 | 5.95 | 9.59 | 32.77 | 51.95 |  | | 44.2 | 3.76 | 5.42 | 14.97 | 25.81 |  | 15.25 | 26.24 |  |  |
| LFR | % | 65.0 | 3.20 | 5.16 | 17.65 | 27.97 |  | | 57.6 | 2.89 | 4.16 | 14.61 | 22.93 |  | 84.8 | 4.72 |  |  |
| MFR | % | 29.7 | 6.71 | 9.06 | 28.80 | 46.92 |  | | 35.7 | 3.89 | 5.73 | 15.43 | 26.89 |  | 4.5 | 50.34 |  |  |
| HFR | % | 5.3 | 14.00 | 20.33 | 55.00 | 95.66 |  | | 6.7 | 15.36 | 16.94 | 43.36 | 77.24 |  | 10.7 | 17.46 |  |  |
| RET-He | pg | 24.7 | 0.46 | 0.67 | -2.35 | 3.69 |  | | 26.2 | 0.49 | 0.62 | -4.90 | 6.14 |  | 26.4 | 1.74 |  |  |
| Abbreviations: 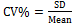 . 100; HCT, hematocrit; HGB, hemoglobin concentration; IPF, immature platelet fraction; IRF, immature reticulocyte fraction; LFR, MFR, and HFR, low-, medium-, and high reticulocyte fluorescence ratio; MCH, mean corpuscular hemoglobin; MCHC, mean corpuscular hemoglobin concentration; MCV, mean corpuscular volume; MPV, mean platelet volume; PCT, plateletcrit; PDW, platelet distribution width; PLT-F, PLT-I, and PLT-O, fluorescence, impedance, and optical platelet counts; P-LCR, platelet large cell ratio; QGI, quality goal index, 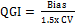; RBC-He, mature RBC hemoglobin equivalent; RBC-I and RBC-O, impedance, and optical RBC counts; RDW-SD and RDW-CV, red cell distribution width standard deviation and coefficient of variation; Repeat, repeatability; Reprod, reproducibility; RET, reticulocyte; RET-He, Reticulocyte hemoglobin equivalent; TE_a_, allowable total error; TE_obs_, observed total error; WBC-D, WBC counted on the WDF channel  *, TE_a_ and TE_obs_ were calculated and reported according to Nabity et al^16^; ^a^, TE_a_ for concentration lower than below the reference interval; ^b^, TE_a_ for concentration within the reference interval; ^c^, TE_a_ for concentration above the reference interval. | | | | | | | | | | | | | | | | | | |

**Table S2.** Comparison of canine blood specimen measurements (n=64) on the Sysmex XT and Sysmex XN-V analyzers (95% confidence intervals of Spearman’s correlation coefficient r and Passing-Bablok equation coefficients between brackets)

|  | Median  (min to max) | | | Spearman’s  r | Passing-Bablok equation  $XN=a.XT+b$ | |
| --- | --- | --- | --- | --- | --- | --- |
|  | XT | XN | XN-XT |  | a | b |
| RBC-I  10^12^/L | 6.13  (2.0 to 9.0) | 6.16  (2.2 to 9.2) | 0.01  (-0.25 to 0.19) | 1.00  (0.99 to 1.00) | 0.99  (0.97 to 1.00) | 0.10  (-0.01 to 0.23) |
| RBC-O  10^12^/L | 6.04  (2.3 to 9.2) | 5.74  (2.1 to 8.5) | -0.30  (-0.73 to 0.05) | 0.99  (0.98 to 0.99) | 0.94  (0.91 to 0.98) | 0.03  (-0.17 to 0.23) |
| HGB  g/L | 143.5  (49 to 217) | 144.0  (50 to 221) | -2.0  (-5.0 to 2.0) | 1.00  (1.00 to 1.00) | 0.99  (0.98 to 1.00) | -0.45  (-2.00 to 1.00) |
| HCT  L/L | 0.400  (0.147 to 0.592) | 0.404  (0.147 to 0.593) | 0.000  (-0.022 to 0.028) | 0.98  (0.97 to 0.99) | 1.02  (0.98 to 1.05) | 0.00  (-0.02 to 0.01) |
| MCV  fL | 66.2  (53.4 to 95.9) | 66.5  (55.5 to 88.3) | 0.8  (-7.6 to 2.8) | 0.97  (0.96 to 0.98) | 0.82  (0.78 to 0.86) | 12.4  (9.83 to 15.56) |
| MCH  pg | 24.1  (19.5 to 27.5) | 23.6  (18.9 to 27.4) | -0.5  (-1.1 to 0.6) | 0.98  (0.96 to 0.99) | 1.13  (1.06 to 1.21) | -3.6  (-5.4 to -1.8) |
| MCHC  g/L | 363  (275 to 398) | 353  (291 to 378) | -11  (-28 to 17) | 0.78  (0.66 to 0.86) | 1.00  (0.80 to 1.18) | -11.00  (-76.40 to 61.80) |
| RDW-SD  fL | 34.7  (30.9 to 75.0) | 31.6  (27.9 to 58.7) | -3.0  (-4.6 to -0.9) | 0.92  (0.87-0.95) | 1.00  (0.91 to 1.06) | -3.00  (-5.25 to 0.11) |
| RDW-CV  % | 15.3  (12.3 to 24.3) | 13.7  (11.6 to 26.1) | -1.7  (-3.5 to 1.8) | 0.96  (0.93 to 0.97) | 0.91  (0.95 to 1.00) | -0.28  (-1.65 to 0.66) |
| RBC-He  pg | 26.1  (20.6 to 30.3) | 23.5  (19.5 to 27.5) | -1.7  (-3.4 to -1.0) | 0.93  (0.88 to 0.96) | 0.92  (0.86 to 1.00) | 0.49  (-1.70 to 1.99) |
|  |  |  |  |  |  |  |
|  |  |  |  |  |  |  |
| PLT-I  10^9^/L | 339.5  (38.0 to 834.0) | 349.5  (20.0 to 994.0) | 19.0  (-36.0 to 116.0) | 0.99  (0.98 to 0.99) | 1.03  (1.00 to 1.06) | 3.32  (-3.25 to 12.80) |
| PLT-O  10^9^/L | 350.5  (34.0 to 894.0) | 379.5  (36.0 to 1019.0) | 36.6  (-22.0 to 129.0) | 0.99  (0.98 to 0.99) | 1.14  (1.10 to 1.17) | -0.86  (-8.93 to 10.73) |
| PDW  fL | 11.3  (8.1 to 17.2) | 11.0  (7.9 to 9.4) | -0.3  (-1.2 to 1.7) | 0.97  (0.94 to 0.98) | 1.08  (1.02 to 1.17) | -1.29  (-2.17 to -0.49) |
| MPV  fL | 10.0  (8.1 to 12.8) | 9.9  (7.8 to 13.5) | 0.4  (-0.9 to 0.6) | 0.96  (0.94 to 0.98) | 0.89  (0.83 to 0.95) | 0.77  (0.10 to 1.33) |
| P-LCR  % | 25.8  (11.0 to 46.4) | 23.1  (8.6 to 46.1) | -2.3  (-6.3 to 2.8) | 0.97  (0.94 to 0.98) | 1.06  (0.99 to 1.11) | -3.97  (-5.45 to -2.12) |
| PCT  mL/L | 3.4  (0.3 to 7.8) | 3.5  (0.3 to 9.0) | 0.0  (-0.6 to 1.2) | 0.98  (0.97 to 0.99) | 0.97  (0.93 to 1.00) | 0.14  (0.00 to 0.26) |
|  |  |  |  |  |  |  |
| WBC¹  10^9^/L | 9.55  (3.03 to 49.05) | 9.13  (2.98 to 46.91) | -0.42  (-2.35 to 0.80) | 0.99  (0.99 to 1.00) | 0.95  (0.93 to 0.97) | 0.03  (-0.17 to 0.24) |
| WBC-D¹  10^9^/L | 9.21  (2.94 to 46.00) | 9.16  (2.90 to 47.21) | 0.15  (-1.45 to 1.76) | 0.99  (0.99 to 1.00) | 1.03  (1.01 to 1.05) | -0.19  (-0.35 to 0 .03) |
| Neutrophils  10^9^/L | 6.42  (2.59 to 43.47) | 6.24  (2.51 to 41.55) | -0.29  (-5.92 to 0.53) | 0.99  (0.99 to 1.00) | 0.94  (0.92 to 0.96) | 0.07  (-0.07 to 0.23) |
| Lymphocytes  10^9^/L | 1.76  (0.16 to 5.99) | 0.15  (0.33 to 6.33) | -0.10  (-0.74 to 4.29) | 0.94  (0.89 to 0.96) | 0.94  (0.86 to 1.02) | 0.00  (-0.15 to 0.13) |
| Monocytes  10^9^/L | 0.65  (0.15 to 3.26) | 0.69  (0.11 to 2.92) | 0.00  (-0.71 to 0.31) | 0.96  (0.93 to 0.97) | 0.90  (0.83 to 1.00) | 0.06  (0.00 to 0.13) |
| Eosinophils  10^9^/L | 0.24  (0.01 to 1.45) | 0.24  (0.01 to 1.38) | -0.00  (-0.17 to 0.14) | 0.98  (0.97 to 0.99) | 0.96  (0.93 to 1.00) | 0.00  (-0.01 to 0.01) |
|  |  |  |  |  |  |  |
| RET count  10^9^/L | 52.6  (4.4 to 270.4) | 47.7  (2.2 to 281.5) | -4.35  (-70.40 to 11.20) | 0.95  (0.92 to 0.97) | 0.88  (0.80 to 0.97) | 1.16  (-2.76 to 4.43) |
| RET  % | 0.85  (0.10 to 12.68) | 0.72  (0.10 to 12.68) | -0.08  (-2.17 to 0.39) | 0.97  (0.95 to 0.98) | 0.02  (-0.06 to 0.08) | 0.87  (0.79 to 0.97) |
| LFR  % | 87.3  (17.4 to 98.2) | 75.6  (35.7 to 96.9) | -11.9  (-31.5 to 18.3) | 0.69  (0.53 to 0.80) | 1.60  (1.26 to 2.00) | -62.88  (-97.70 to -34.41) |
| MFR  % | 9.3  (0.0 to 14.1) | 11.8  (0.0 to 15.1) | 2.2  (-14.1 to 17.1) | 0.43  (0.20 to 0.61) | 0.80  (0.56 to 1.19) | 3.73  (1.27 to 6.05) |
| HFR  % | 3.4  (0.7 to 13.8) | 11.5  (0.8 to 35.9) | 8.4  (-3.5 to 31.5) | 0.34  (0.10 to 0.54) | 5.24  (3.47 to 9.07) | -5.72  (-19.16 to -0.29) |
| IRF  % | 12.7  (1.8 to 82.6) | 24.4  (3.1 to 64.3) | 11.9  (-18.3 to 31.5) | 0.69  (0.53 to 0.80) | 1.61  (1.26 to 2.00) | 2.62  (-2.34 to 8.25) |
| RET-He  pg | 27.3  (19.6 to 30.6) | 23.1  (17.2 to 26.9) | -3.1  (-6.8 to 0.0) | 0.80  (0.69 to 0.88) | 0.86  (0.74 to 0.96) | 0.91  (-1.92 to 3.87) |
| Abbreviations: HCT, hematocrit; HGB, hemoglobin concentration; IRF, immature reticulocyte fraction; LFR, MFR, and HFR, low-, medium-, and high reticulocyte fluorescence ratio; MCH, mean corpuscular hemoglobin; MCHC, mean corpuscular hemoglobin concentration; MCV, mean corpuscular volume; max, maximum; min, minimum; MPV, mean platelet volume; PCT, plateletcrit; PDW, platelet distribution width; PLT-I and PLT-O, impedance, and optical platelet counts; P-LCR, platelet large cell ratio; RBC-He, mature RBC hemoglobin equivalent; RBC-I and RBC-O, impedance, and optical RBC counts; RDW-SD and RDW-CV, red cell distribution width standard deviation and coefficient of variation; RET, reticulocyte; RET-He, Reticulocyte hemoglobin equivalent; WBC-D, WBC counted on the WDF channel.  ¹ One pair of outliers deleted (WBC_XT_: 209.10^9^/L; WBC_XN_: 192.10^9^/L) | | | | | | |
|  | | | | | | |

**Table S3.** Classification of hematology measurements (n= 64) with the Sysmex XT-2000iV and Sysmex XN-V analyzers according to canine Sysmex XT-2000iV reference intervals.^14^

|  |  |  | Classification according to XT’s RIs | | |
| --- | --- | --- | --- | --- | --- |
|  |  |  | <LRL | WRI | >URL |
| RBC-I | XT results  nb of XN differences |  | 12  +1* | 46 | 6  -1* |
| RBC-O | XT results  nb of XN differences |  | 13  +5* | 45 | 6  +2* |
| HGB | XT results  nb of XN differences |  | 13  +2* | 48 | 3  0 |
| HCT | XT results  nb of XN differences |  | 13  +1* | 48 | 3  +1* |
| MCV | XT results  nb of XN differences |  | 4  +1+1* | 52 | 8  +1+1* |
| MCH | XT results  nb of XN differences |  | 3  +5* | 58 | 3  +1* |
| MCHC | XT results  nb of XN differences |  | 6  +9*-1 | 53 | 5  -5 |
| RDW-SD | XT results  nb of XN differences |  | 1  +25* | 56 | 7  -2-1* |
| RDW-CV | XT results  nb of XN differences |  | 2  +10+14* | 58 | 4  -1 |
| RBC-He | XT results  nb of XN differences |  |  | RI na |  |
|  |  |  |  |  |  |
| PLT-I | XT results  nb of XN differences |  | 6  0 | 52 | 6  -1*+1* |
| PLT-O | XT results  nb of XN differences |  | 4  0 | 48 | 9  +3* |
| PDW | XT results  nb of XN differences |  | 6  +5* | 48 | 0  0 |
| MPV | XT results  nb of XN differences |  | 7  +3 | 47 | 1  -1* |
| P-LCR | XT results  nb of XN differences |  | 8  +1+5* | 47 | 0  0 |
| PCT | XT results  nb of XN differences |  | 2  0 | 46 | 7  0 |
|  |  |  |  |  |  |
| WBC^1^ | XT results  nb of XN differences |  | 5  +1 | 54 | 4  -1* |
| WBC-D^1^ | XT results  nb of XN differences |  |  | RI na |  |
| Neutrophils | XT results  nb of XN differences |  | 2  0 | 48 | 9  -1 |
| Lymphocytes | XT results  nb of XN differences |  | 15  +3* | 43 | 1  +1 |
| Monocytes | XT results  nb of XN differences |  | 9  +1*-2* | 45 | 7 |
| Eosinophils | XT results  nb of XN differences |  | 16  -1*+1* | 48 | 0  0 |
|  |  |  |  |  |  |
| RET count | XT results  nb of XN differences |  | 7  +1+3* | 54 | 3  0 |
| RET percentage | XT results  nb of XN differences |  | 6  +1* | 52 | 5  -1 |
| LFR | XT results  nb of XN differences |  | 1  +8*+4 | 58 | 5  -4 |
| MFR | XT results  nb of XN differences |  | 10  -9+2 | 52 | 2  -2 |
| HFR | XT results  nb of XN differences |  | 5  -5 | 58 | 1  -1* |
| IRF | XT results  nb of XN differences |  | 6  -5 | 57 | 1  +6*+5 |
| RET-He | XT results  nb of XN differences |  |  | RIs na |  |
|  |  |  |  |  |  |
| Note: For XT, the number of cases within the reference interval (WRI), lower or higher than the reference limits (LRL and URL); for XN, number of differences from XT classification.  Abbreviations: HCT, hematocrit; HGB, hemoglobin concentration; IRF, immature reticulocyte fraction; LFR, MFR, and HFR, low-, medium-, and high reticulocyte fluorescence ratio; LRL, lower reference limits; MCH, mean corpuscular hemoglobin; MCHC, mean corpuscular hemoglobin concentration; MCV, mean corpuscular volume; MPV, mean platelet volume; PCT, plateletcrit; PDW, platelet distribution width; nb, number; PLT-I and PLT-O, impedance, and optical platelet counts; P-LCR, platelet large cell ratio; RBC-He, mature RBC hemoglobin equivalent; RBC-I and RBC-O, impedance, and optical RBC counts; RDW-SD and RDW-CV, red cell distribution width standard deviation and coefficient of variation; RET, reticulocyte; RET-He, Reticulocyte hemoglobin equivalent; RIs, reference intervals; RIs na, RIs not available; URI, upper reference limits; WBC-D, WBC counted on the WDF channel; WRI: within the RI.  *, number of cases within the 90% CI of XT reference limits.  ¹ One pair of outliers deleted (WBC_XT_: 209.10^9^/L; WBC_XN_: 192.10^9^/L). | | | | | |
|  |  |  |  | | |

**Table S4:** Percent change in the Sysmex XN-V measurements for 10 canine EDTA blood specimens according to temperature and duration of storage.

| **Variable** | **T_0_** | **Temperature** | |  | **Effects of storage** (hrs): comparison to T0 | | | | | | |
| --- | --- | --- | --- | --- | --- | --- | --- | --- | --- | --- | --- |
|  |  | **P** |  |  | **P** | **2** | **4** | **8** | **24** | **48** | **72** |
| **RBC-I** | 3.92 to 7.55  10^12^/L | <0.001 | 4 |  | 0.008 | 100 | 99 | 100 | 100 | 99 | 99 |
|  |  |  |  |  |  | *0.994* | *0.738* | *0.874* | *1.000* | *0.087* | *0.358* |
|  |  |  | 24 |  | 0.998 | 100 | 100 | 99 | 99 | 97 | 96 |
|  |  |  |  |  |  | *0.997* | *0.967* | *0.873* | *0.183* | *<0.001* | *<0.001* |
| **RBC-0** | 3.68 to 6.99  10^12^/L | 0.287 | 4 |  | 0.233 | 100 | 100 | 100 | 100 | 100 | 101 |
|  |  |  |  |  |  | - | - | - | - | - | - |
|  |  |  | 24 |  | 0.008 | 100 | 100 | 100 | 100 | 100 | 100 |
|  |  |  |  |  |  | *0.993* | *0.985* | *1.000* | *0.274* | *0.307* | *0.843* |
| **HGB** | 59.6 to 172.0  g/L | 0.208 | 4 |  | <0.001 | 100 | 100 | 100 | 101 | 101 | 101 |
|  |  |  |  |  |  | *0.997* | *0.957* | *0.457* | ***<0.001*** | ***<0.001*** | ***<0.001*** |
|  |  |  | 24 |  | <0.001 | 100 | 100 | 100 | 101 | 101 | 101 |
|  |  |  |  |  |  | *1.000* | *0.987* | *0.358* | ***0.029*** | ***<0.001*** | ***<0.001*** |
| **HCT** | 0.213 to. 0.499  L/L | <0.001 | 4 |  | <0.001 | 100 | 99 | 100 | 103 | 106 | 108 |
|  |  |  |  |  |  | *0.988* | *0.844* | *1.000* | ***<0.001*** | ***<0.001*** | ***<0.001*** |
|  |  |  | 24 |  | <0.001 | 100 | 101 | 102 | 108 | 113 | 118 |
|  |  |  |  |  |  | *0.849* | *0.536* | ***<0.001*** | ***<0.001*** | ***<0.001*** | ***<0.001*** |
| **MCV** | 48.5 to 72.2  fL | <0.001 | 4 |  | <0.001 | 100 | 100 | 100 | 103 | 107 | 109 |
|  |  |  |  |  |  | *1.000* | *1.000* | *0.797* | ***<0.001*** | ***<0.001*** | ***<0.001*** |
|  |  |  | 24 |  | <0.001 | 101 | 101 | 103 | 110 | 118 | 125 |
|  |  |  |  |  |  | *0.647* | *0.227* | ***<0.001*** | ***<0.001*** | ***<0.001*** | ***<0.001*** |
| **MCH** | 13.6 to 24.5  pg | <0.001 | 4 |  | <0.001 | 100 | 101 | 101 | 101 | 102 | 102 |
|  |  |  |  |  |  | *0.989* | *0.397* | *0.435* | *0.186* | ***<0.001*** | ***<0.001*** |
|  |  |  | 24 |  | <0.001 | 100 | 100 | 101 | 102 | 105 | 106 |
|  |  |  |  |  |  | *0.993* | *0.890* | *0.629* | ***0.025*** | ***<0.001*** | ***<0.001*** |
| **MCHC** | 281 to 359  g/L | <0.001 | 4 |  | <0.001 | 100 | 101 | 100 | 98 | 95 | 93 |
|  |  |  |  |  |  | *0.999* | *0.909* | *1.000* | ***0.004*** | ***<0.001*** | ***<0.001*** |
|  |  |  | 24 |  | <0.001 | 99 | 99 | 98 | 93 | 89 | 85 |
|  |  |  |  |  |  | *0.973* | *0.831* | ***0.026*** | ***<0.001*** | ***<0.001*** | ***<0.001*** |
| **RDW-SD** | 29.1 to 35.6  fL | <0.001 | 4 |  | <0.001 | 100 | 100 | 101 | 105 | 111 | 115 |
|  |  |  |  |  |  | *1.000* | *0.998* | *0.989* | ***<0.001*** | ***<0.001*** | ***<0.001*** |
|  |  |  | 24 |  | <0.001 | 102 | 101 | 104 | 112 | 124 | 135 |
|  |  |  |  |  |  | *0.767* | *0.941* | ***0.002*** | ***<0.001*** | ***<0.001*** | ***<0.001*** |
| **RDW-CV** | 11.6 to 23.2  % | <0.001 | 4 |  | <0.001 | 100 | 100 | 100 | 102 | 104 | 107 |
|  |  |  |  |  |  | *1.000* | *1.000* | *0.999* | *0.811* | ***0.008*** | ***<0.001*** |
|  |  |  | 24 |  | 0.043 | 100 | 101 | 100 | 98 | 98 | 99 |
|  |  |  |  |  |  | *1.000* | *0.999* | *0.999* | *0.405* | *0.238* | *0.861* |
| **NRBC** | 0.0 to 0.39  10^9^/L | 0.451 | 4 |  | 0.003 | 99 | 95 | 100 | 112 | 124 | 123 |
|  |  |  |  |  |  | *0.999* | *1.000* | *0.998* | *0.517* | ***0.038*** | *0.051* |
|  |  |  | 24 |  | 0.001 | 91 | 109 | 107 | 126 | 145 | 102 |
|  |  |  |  |  |  | *0.991* | *1.000* | *0.998* | *0.385* | ***0.012*** | *1.000* |
| **RBC-He** | 14.7 to 26.1  pg | 0.001 | 4 |  | <0.001 | 100 | 100 | 100 | 99 | 98 | 97 |
|  |  |  |  |  |  | *0.747* | *0.905* | *0.336* | ***<0.001*** | ***<0.001*** | ***<0.001*** |
|  |  |  | 24 |  | <0.001 | 100 | 99 | 100 | 99 | 97 | 95 |
|  |  |  |  |  |  | *0.998* | *0.820* | *1.000* | *0.068* | ***<0.001*** | ***<0.001*** |
| **WBC** | 6.69 to 29.06  10^9^/L | <0.001 | 4 |  | 0.143 | 101 | 101 | 101 | 101 | 101 | 102 |
|  |  |  |  |  |  | - | - | - | - | - | - |
|  |  |  | 24 |  | <0.001 | *99* | *103* | *99* | *99* | *97* | *95* |
|  |  |  |  |  |  | *0.954* | *0.400* | *0.958* | *0.252* | ***<0.001*** | ***<0.001*** |
| **WBC-D** | 5.95 to 29.45  10^9^/L | <0.001 | 4 |  | 0.143 | 100 | 101 | 101 | 101 | 103 | 103 |
|  |  |  |  |  |  | - | - | - | - | - | - |
|  |  |  | 24 |  | <0.001 | 99 | 103 | 100 | 100 | 97 | 91 |
|  |  |  |  |  |  | *0.991* | *0.942* | *1.000* | *1.000* | ***0.023*** | ***<0.001*** |

| **Neutrophils^1^** | 2.87 to 13.91  10^9^/L | <0.001 | 4 |  | <0.001 | 100 | 102 | 101 | 102 | 104 | 107 |
| --- | --- | --- | --- | --- | --- | --- | --- | --- | --- | --- | --- |
|  |  |  |  |  |  | *0,890* | *0,960* | *0,915* | ***0,050*** | ***<0,001*** | ***<0,001*** |
|  |  |  | 24 |  | 0.585 | 100 | 99 | 99 | 99 | 101 | 101 |
|  |  |  |  |  |  | - | - | - | - | - | - |
| **Lymphocytes^1^** | 1.49 to 4.82  10^9^/L | 0.051 | 4 |  | <0.001 | 100 | 101 | 101 | 101 | 95 | 90 |
|  |  |  |  |  |  | *1,000* | *1,000* | *1,000* | *0,651* | *0,057* | ***0,003*** |
|  |  |  | 24 |  | 0.118 | 100 | 100 | 99 | 100 | 96 | 94 |
|  |  |  |  |  |  | - | - | - | - | - | - |
| **Monocytes^1^** | 0.38 to 1.38  10^9^/L | <0.001 | 4 |  | 0.077 | 100 | 100 | 101 | 97 | 95 | 95 |
|  |  |  |  |  |  | - | - | - | - | - | - |
|  |  |  | 24 |  | <0.001 | 100 | 97 | 100 | 98 | 89 | 57 |
|  |  |  |  |  |  | *1,000* | *1,000* | *1,000* | *0,671* | ***<0,001*** | ***<0,001*** |
| **Eosinophils^1^** | 0.40 to 1.87  10^9^/L | 0.773 | 4 |  | 0.220 | 103 | 102 | 104 | 103 | 101 | 103 |
|  |  |  |  |  |  | - | - | - | - | - | - |
|  |  |  | 24 |  | 0.003 | 102 | 103 | 103 | 102 | 106 | 110 |
|  |  |  |  |  |  | *0,922* | *0,889* | *0,818* | *0,962* | *0,109* | ***0,002*** |

| **PLT-I** | 227 to 819  10^9^/L | <0.001 | 4 |  | <0.001 | 93 | 92 | 86 | 70 | 58 | 53 |
| --- | --- | --- | --- | --- | --- | --- | --- | --- | --- | --- | --- |
|  |  |  |  |  |  | *0.667* | *0.581* | ***0.044*** | ***<0.001*** | ***<0.001*** | ***<0.001*** |
|  |  |  | 24 |  | <0.001 | 95 | 92 | 88 | 81 | 72 | 69 |
|  |  |  |  |  |  | *0.886* | *0.342* | *0.057* | ***<0.001*** | ***<0.001*** | ***<0.001*** |
| **PLT-F** | 240 to 882  10^9^/L | 0.513 | 4 |  | <0.001 | 94 | 95 | 90 | 83 | 72 | 65 |
|  |  |  |  |  |  | *0.881* | *0.794* | *0.178* | ***<0.001*** | ***<0.001*** | ***<0.001*** |
|  |  |  | 24 |  | <0.001 | 97 | 90 | 91 | 85 | 68 | 59 |
|  |  |  |  |  |  | *1.000* | *0.992* | *0.911* | *0.417* | ***<0.001*** | ***<0.001*** |
| **PLT-O** | 255 to 933  10^9^/L | 0.016 | 4 |  | <0.001 | 95 | 91 | 88 | 82 | 73 | 72 |
|  |  |  |  |  |  | *0.977* | *0.671* | *0.303* | ***0.009*** | ***<0.001*** | ***<0.001*** |
|  |  |  | 24 |  | <0.001 | 96 | 90 | 92 | 88 | 80 | 79 |
|  |  |  |  |  |  | *0.829* | *0.635* | *0.214* | ***0.013*** | ***<0.001*** | ***<0.001*** |
| **PDW** | 10.1 to 13.4  fL | 0.059 | 4 |  | <0.001 | 98 | 104 | 107 | 108 | 113 | 125 |
|  |  |  |  |  |  | *0.855* | *0.737* | *0.102* | ***<0.001*** | ***<0.001*** | ***<0.001*** |
|  |  |  | 24 |  | <0.001 | 101 | 102 | 105 | 113 | 122 | 114 |
|  |  |  |  |  |  | *1.000* | *0.998* | *0.660* | ***<0.001*** | ***<0.001*** | ***<0.001*** |
| **PCT** | 2.4 to 8.3  mL/L | <0.001 | 4 |  | <0.001 | 94 | 93 | 88 | 74 | 62 | 58 |
|  |  |  |  |  |  | *0.827* | *0.644* | *0.099* | ***<0.001*** | ***<0.001*** | ***<0.001*** |
|  |  |  | 24 |  | <0.001 | 95 | 90 | 89 | 87 | 80 | 73 |
|  |  |  |  |  |  | *0.881* | *0.246* | *0.116* | ***0.045*** | ***<0.001*** | ***<0.001*** |
| **MPV** | 9.3 to 12.1  fL | 0.934 | 4 |  | <0.001 | 102 | 101 | 103 | 105 | 106 | 109 |
|  |  |  |  |  |  | *0.596* | *0.912* | *0.064* | ***0.002*** | ***<0.001*** | ***<0.001*** |
|  |  |  | 24 |  | <0.001 | 100 | 101 | 101 | 108 | 111 | 107 |
|  |  |  |  |  |  | *1.000* | *0.941* | *1.000* | ***<0.001*** | ***<0.001*** | ***<0.001*** |
| **P-LCR** | 19.1 to 44.9  % | 0.860 | 4 |  | <0.001 | 108 | 105 | 112 | 117 | 122 | 131 |
|  |  |  |  |  |  | *0.349* | *0.769* | *0.029* | ***<0.001*** | ***<0.001*** | ***<0.001*** |
|  |  |  | 24 |  | <0.001 | 99 | 97 | 103 | 127 | 136 | 124 |
|  |  |  |  |  |  | *1.000* | *0.987* | *0.988* | ***<0.001*** | ***<0.001*** | ***<0.001*** |
| **IPF** | 2.25 to 9.75  % | 0.011 | 4 |  | <0.001 | 103 | 105 | 111 | 154 | 178 | 177 |
|  |  |  |  |  |  | *1.000* | *1.000* | *0.996* | *0.053* | ***0.001*** | ***0.001*** |
|  |  |  | 24 |  | <0.001 | 104 | 104 | 108 | 119 | 145 | 158 |
|  |  |  |  |  |  | *1.000* | *1.000* | *0.987* | *0.572* | ***0.002*** | ***<0.001*** |
| **RET** | 23.0 to 194.7  10^9^/L | 0.004 | 4 |  | <0.001 | 99 | 98 | 98 | 104 | 103 | 109 |
|  |  |  |  |  |  | *0.998* | *0.999* | *1.000* | *0.461* | *0.883* | ***<0.001*** |
|  |  |  | 24 |  | <0.001 | 100 | 102 | 99 | 101 | 110 | 119 |
|  |  |  |  |  |  | *1.000* | *1.000* | *0.999* | *1.000* | *0.147* | ***0.002*** |
| **RET** | 0.32 to 4.96  % | 0.003 | 4 |  | <0.001 | 99 | 98 | 99 | 104 | 103 | 109 |
|  |  |  |  |  |  | *1.000* | *1.000* | *1.000* | *0.545* | *0.890* | ***<0.001*** |
|  |  |  | 24 |  | <0.001 | 101 | 103 | 100 | 102 | 114 | 124 |
|  |  |  |  |  |  | *1.000* | *1.000* | *1.000* | *1.000* | *0.139* | ***0.007*** |
| **IRF** | 17.3 to 53.2  % | 0.423 | 4 |  | 0.260 | 100 | 100 | 101 | 97 | 107 | 101 |
|  |  |  |  |  |  | - | - | - | - | - | - |
|  |  |  | 24 |  | 0.432 | 102 | 106 | 102 | 105 | 101 | 98 |
|  |  |  |  |  |  | - | - | - | - | - | - |
| **LFR** | 46.9 to 82.7  % | 0.423 | 4 |  | 0.260 | 101 | 101 | 102 | 104 | 100 | 102 |
|  |  |  |  |  |  | - | - | - | - | - | - |
|  |  |  | 24 |  | 0.432 | 99 | 99 | 99 | 98 | 101 | 102 |
|  |  |  |  |  |  | - | - | - | - | - | - |
| **MFR** | 5.8 to 25.6  % | <0.001 | 4 |  | 0.181 | 105 | 102 | 108 | 103 | 109 | 100 |
|  |  |  |  |  |  | - | - | - | - | - | - |
|  |  |  | 24 |  | 0.005 | 100 | 96 | 95 | 95 | 94 | 88 |
|  |  |  |  |  |  | *1.000* | *0.812* | *0.636* | *0.756* | *0.402* | ***0.004*** |
| **HFR** | 7.3 to 34.4  % | <0.001 | 4 |  | 0.010 | 92 | 93 | 88 | 86 | 94 | 95 |
|  |  |  |  |  |  | *0.384* | *0.558* | *0.040* | *0.005* | *0.721* | *0.785* |
|  |  |  | 24 |  | 0.689 | 103 | 105 | 107 | 109 | 103 | 102 |
|  |  |  |  |  |  | - | - | - | - | - | - |
| **RET-He** | 14.8 to 26.5  pg | <0.001 | 4 |  | <0.001 | 99 | 99 | 100 | 98 | 96 | 96 |
|  |  |  |  |  |  | *0.977* | *0.999* | *1.000* | *0.748* | ***0.003*** | ***<0.001*** |
|  |  |  | 24 |  | <0.001 | 100 | 98 | 100 | 101 | 99 | 97 |
|  |  |  |  |  |  | *1.000* | *1.000* | *1.000* | *0.875* | *0.350* | ***<0.001*** |
| Note: T_0_, range of measurements at T_0_; P, ANOVA of the effects of temperature and duration of storage; 2 to 72, effects of time: mean of percent of T_0_; in italics, comparison to T_0_ by Tukey’s HSD test.  Abbreviations: HCT, hematocrit; HGB, hemoglobin concentration; IPF, immature platelet fraction; IRF, immature reticulocyte fraction; LFR, MFR, and HFR, low-, medium-, and high reticulocyte fluorescence ratio; MCH, mean corpuscular hemoglobin; MCHC, mean corpuscular hemoglobin concentration; MCV, mean corpuscular volume; MPV, mean platelet volume; NRBC, nucleated red blood cells; PCT, plateletcrit; PDW, platelet distribution width; PLT-F, PLT-I, and PLT-O, fluorescence, impedance, and optical platelet counts; P-LCR, platelet large cell ratio; RBC-He, mature RBC hemoglobin equivalent; RBC-I, RBC-O, impedance, and optical RBC counts; RDW-SD and RDW-CV, red cell distribution width standard deviation and coefficient of variation; RET, reticulocyte; RET-He, Reticulocyte hemoglobin equivalent.  ^1^ Two outliers deleted (abnormal WBC scattergrams with arbitrary clouds separation). | | | | | | | | | | | |
