# Supplementary material for: Validation of the Sysmex XN‐V hematology analyzer for canine specimens
Source: Vet Clin Pathol. 2021 Jun 21;50(2):184–97. doi: 10.1111/vcp.12936 (PMC8362000; doi:10.1111/vcp.12936)
Supplement: Supplementary file 6 — Supplementary Material [file VCP-50-184-s005.docx]

**Figure S1.** Measurements of hematology variables expressed as blood concentrations or counts with the Sysmex XN-V analyzer after dilution (concentration of undiluted specimen normalized to 1).

**Abbreviations:** HCT, hematocrit; HGB, hemoglobin concentration; PCT, plateletcrit; PLT-F, PLT-I, and PLT-O, fluorescence, impedance, and optical platelet counts; RBC-I and RBC-O, impedance, and optical RBC counts; RET, reticulocytes; WBC-D, WBC counted on the WDF channel.

**Figure S2.** Passing-Bablok plots (left) and difference diagrams (right) from comparison between the Sysmex XN-V and the Sysmex XT-2000iV measurements. In Passing-Bablok plots, the thin gray line is identity (y=x); the blue line is the regression curve with 95% confidence intervals.

**Abbreviations:** HCT, hematocrit; HGB, hemoglobin concentration; IPF, immature platelet fraction; IRF, immature reticulocyte fraction; LFR; MFR and HFR, low-, medium-, and high reticulocyte fluorescence ratio; MCH, mean corpuscular hemoglobin; MCHC, mean corpuscular hemoglobin concentration; MCV, mean corpuscular volume; MPV, mean platelet volume; PCT, plateletcrit; PDW, platelet distribution width; PLT-I and PLT-O, impedance, and optical platelet counts; P-LCR, platelet large cell ratio; RBC-He, mature RBC hemoglobin equivalent; RBC-I and RBC-O, impedance, and optical RBC counts; RDW-SD and RDW-CV, red cell distribution width standard deviation and coefficient of variation; RET, reticulocyte; RET-He, Reticulocyte hemoglobin equivalent; WBC-D, WBC counted on the WDF channel.

**Figure S3**. Effects of hemolysis (blue), lipemia (orange), and icterus (gray) on canine hematology variables with the Sysmex XN-V (maximum concentrations of hemoglobin: 5 g/L; triglycerides: 10 g/L; bilirubin: 0.15 g/L; changes as % difference from native specimen); a, b, c: comparison to native specimen using Dunnett’s test, *P*<0.05; 0.01; 0.001.

**Abbreviations:** HCT, hematocrit; HGB, hemoglobin concentration; IPF, immature platelet fraction; IRF, immature reticulocyte fraction; LFR; MFR and HFR, low-, medium-, and high reticulocyte fluorescence ratio; MCH, mean corpuscular hemoglobin; MCHC, mean corpuscular hemoglobin concentration; MCV, mean corpuscular volume; MPV, mean platelet volume; PCT, plateletcrit; PDW, platelet distribution width; PLT-I and PLT-O, impedance, and optical platelet counts; P-LCR, platelet large cell ratio; RBC-He, mature RBC hemoglobin equivalent; RBC-I and RBC-O, impedance, and optical RBC counts; RDW-SD and RDW-CV, red cell distribution width standard deviation and coefficient of variation; RET, reticulocyte; RET-He, Reticulocyte hemoglobin equivalent; WBC-D, WBC counted on the WDF channel.

**Figure S4.** Effects of carry-over on hematology measurements with the Sysmex XN-V (Five repeats of manufacturer’s high and low control solutions).

**Abbreviations:** HCT, hematocrit; HGB, hemoglobin concentration; IPF, immature platelet fraction; IRF, immature reticulocyte fraction; LFR; MFR and HFR, low-, medium-, and high reticulocyte fluorescence ratio; MCH, mean corpuscular hemoglobin; MCHC, mean corpuscular hemoglobin concentration; MCV, mean corpuscular volume; MPV, mean platelet volume; NRBC, nucleated RBC count; PCT, plateletcrit; PDW, platelet distribution width; PLT-F, PLT-I, and PLT-O, fluorescence, impedance, and optical platelet counts; P-LCR, platelet large cell ratio; RBC-He, mature RBC hemoglobin equivalent; RBC-I and RBC-O, impedance, and optical RBC counts; RDW-SD and RDW-CV, red cell distribution width standard deviation and coefficient of variation; RET, reticulocyte; RET-He, Reticulocyte hemoglobin equivalent; WBC-D, WBC counted on the WDF channel.
